# Loss of OVOL2 in Triple‐Negative Breast Cancer Promotes Fatty Acid Oxidation Fueling Stemness Characteristics

**DOI:** 10.1002/advs.202308945

**Published:** 2024-04-16

**Authors:** Ruipeng Lu, Jingjing Hong, Tong Fu, Yu Zhu, Ruiqi Tong, Di Ai, Shuai Wang, Qingsong Huang, Ceshi Chen, Zhiming Zhang, Rui Zhang, Huiling Guo, Boan Li

**Affiliations:** ^1^ State Key Laboratory of Cellular Stress Biology School of Life Sciences Xiamen University Xiamen 361104 China; ^2^ Academy of Biomedical Engineering Kunming Medical University Kunming 650500 China; ^3^ The Third Affiliated Hospital Kunming Medical University Kunming 650118 China; ^4^ Department of Breast Surgery The First Affiliated Hospital of Xiamen University Xiamen 361009 China; ^5^ Xiamen Cell Therapy Research Center The First Affiliated Hospital of Xiamen University School of Medicine Xiamen University Xiamen 361003 China

**Keywords:** FAO, JAK/STAT3, Ovol2, stemness characteristics, TNBC

## Abstract

Triple‐negative breast cancer (TNBC), the most aggressive subtype of breast cancer, has a poor prognosis and lacks effective treatment strategies. Here, the study discovered that TNBC shows a decreased expression of epithelial transcription factor ovo‐like 2 (OVOL2). The loss of OVOL2 promotes fatty acid oxidation (FAO), providing additional energy and NADPH to sustain stemness characteristics, including sphere‐forming capacity and tumor initiation. Mechanistically, OVOL2 not only suppressed STAT3 phosphorylation by directly inhibiting JAK transcription but also recruited histone deacetylase 1 (HDAC1) to STAT3, thereby reducing the transcriptional activation of downstream genes carnitine palmitoyltransferase1 (CPT1A and CPT1B). PyVT‐Ovol2 knockout mice develop a higher number of primary breast tumors with accelerated growth and increased lung‐metastases. Furthermore, treatment with FAO inhibitors effectively reduces stemness characteristics of tumor cells, breast tumor initiation, and metastasis, especially in OVOL2‐deficient breast tumors. The findings suggest that targeting JAK/STAT3 pathway and FAO is a promising therapeutic strategy for OVOL2‐deficient TNBC.

## Graphical abstract

Triple‐negative breast cancer (TNBC), the most aggressive breast cancer subtype, exhibits downregulated expression of the epithelial transcription factor OVOL2. OVOL2 suppresses JAK transcription, recruits HDAC1 and inhibits STAT3 activation, reducing downstream gene (CPT1A and CPT1B) expression. OVOL2 loss enhances fatty acid oxidation (FAO), fueling stemness traits and tumor initiation. Targeting JAK/STAT3 and FAO is a promising strategy for OVOL2‐deficient TNBC.

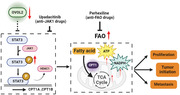

## 1 Introduction

Triple‐negative breast cancer (TNBC), characterized by the absence of estrogen receptor (ER), progesterone receptor (PR), and human epidermal growth factor receptor 2 (HER2), is the most aggressive subtype accounting for 10–20% of all breast cancer cases.^[^ ^1^ ^]^ It has a poor prognosis and a high risk of recurrence. The presence of highly stemness characteristics in TNBC contributes to rapid oncogenic growth and distant metastasis.^[^ ^2^ ^]^ Traditional hormone‐targeted therapies and HER2‐targeted therapies are ineffective in treating TNBC due to the absence of ER, PR, and HER2 receptors. Chemotherapy remains the primary option, albeit with limited effectiveness.^[^ ^3^, ^4^ ^]^ Therefore, it is essential to develop new strategies for TNBC therapy.

Fatty acid β‐oxidation (FAO) plays a crucial role in the metabolism of cancer cells, providing a substantial amount of energy essential for sustaining rapid cell growth and tumor progression. Moreover, FAO generates abundant antioxidants, such as NADPH, which promotes the survival of tumor cells.^[^ ^5^ ^]^ The emerging roles of FAO in cancer progression, chemotherapy resistance, and the maintenance of cancer stem cells (CSCs) have gained significant attention. Inhibition of FAO has been proposed as a potential therapeutic approach to counteract tumor growth in breast cancer.^[^ ^6^, ^7^ ^]^ However, the specific regulatory mechanisms of FAO in TNBC remain unclear.

The signal transducer and activator of transcription 3 (STAT3) is one of the seven members of the STAT family. Canonical STAT3 signaling plays a crucial role in numerous biological processes, including cell proliferation, survival, differentiation, and angiogenesis.^[^ ^8^, ^9^ ^]^ IL‐6 and diverse cytokines exert actions via the transmembrane receptor gp130, triggering Janus kinases (JAKs)/STAT3 signaling.^[^ ^10^ ^]^ Subsequently, STAT3 is recruited to the activated cytokine receptor and phosphorylated by receptor‐associated JAK at tyrosine. Phosphorylated STAT3 could form a dimer, translocate into the nucleus, and ultimately activate transcriptional of downstream genes.^[^ ^11^, ^12^ ^]^ Recent clinical and preclinical data revealed that overexpression and constitutively activation of STAT3 were involved in the progression, proliferation, metastasis, and chemoresistance of breast cancer.^[^ ^13^ ^]^ Moreover, JAK/STAT3 activates transcription of carnitine palmitoyl transferase 1B (CPT1B) facilitating FAO which is crucial for breast cancer stem cell renewal and chemoresistance.^[^ ^14^ ^]^ STAT3 is persistently activated in the context primarily linked to triple‐negative tumors,^[^ ^15^ ^]^ however the precise mechanistic by which sustained STAT3 activation in TNBC remains largely unknown.

Ovo‐like 2 (OVOL2), a transcription repressor factor, plays a vital role in cell differentiation, epithelial‐mesenchymal transition (EMT), and tissue development. Down‐regulation of OVOL2 has been found in many kinds of cancers.^[^ ^16^, ^17^, ^18^ ^]^ We previously found that OVOL2 loss is associated with malignant progression in colorectal cancer.^[^ ^19^ ^]^ OVOL2 suppresses the TGFβ1‐induced EMT process in breast cancer.^[^ ^20^ ^]^ However, the role of OVOL2 in regulating fatty acid oxidation, particularly in TNBC, remains largely unknown.

Here, we have observed a significant decrease in OVOL2 expression in TNBC, which is intricately linked to the persistent activation of STAT3. RNA‐seq analysis revealed that OVOL2 inhibits fatty acid oxidation via STAT3 signaling. Furthermore, we demonstrated that OVOL2 directly suppresses STAT3 phosphorylation by directly inhibiting JAK transcription, recruits HDAC1 to STAT3, and subsequently reduces expressions of CPT1A and CPT1B. Consistently, OVOL2 loss promotes FAO and enhances the production of ATP and NADPH that support the stem cell‐like characteristics of TNBC. In a mouse model, PyVT‐Ovol2 knockout mice developed a higher number of primary breast tumors with increased stemness properties which revealed accelerated growth and elevated lung‐metastases. Our findings uncover the regulatory mechanism by which OVOL2 represses FAO in breast cancer and propose that targeting JAK/STAT3‐FAO is a potential strategy for OVOL2‐absent TNBC therapy.

## 2 Result

### 2.1 OVOL2 Loss in TNBC Promotes Sphere Formation and Tumor Initiation

**Figure 1:**
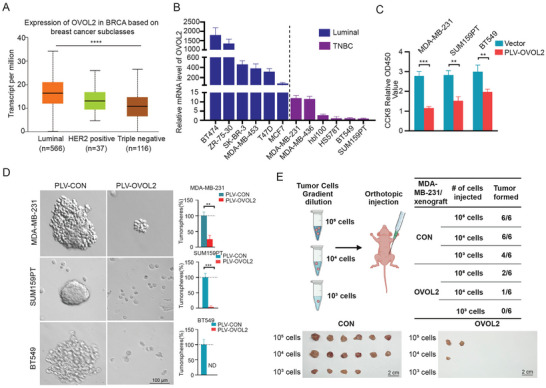
OVOL2 loss in TNBC promotes sphere formation and tumor initiation. A) Boxplots illustrating the expression levels of OVOL2 obtained from the UALCAN database. B) The expression of OVOL2 in 12 human breast cancer cell lines was determined by RT–qPCR. C) Cell growth was evaluated using a CCK8 assay. Data were analyzed 48 h after transfection. D) The ability of OVOL2‐overexpressing cancer cells to induce mammosphere formation was determined by a mammosphere formation assay. The graphs show the sphere numbers compared to those in the control group. E) In vivo tumor formation in mice injected with serial dilutions of MDA‐MB‐231 cells. *p*‐value determined by Student's *t*‐test. The data are presented as means ± SD. The symbols *, **, *** and represent *p* < 0.05, *p* < 0.01 and *p* < 0.001, respectively.

We previously found that mRNA levels of OVOL2 decreased at a late stage of human breast cancer progression.^[^ ^20^ ^]^ To further elucidate the impact of OVOL2 on breast cancer carcinogenicity, we analyzed OVOL2 expression across different molecular subtypes using data from UALCAN and Oncomine databases. OVOL2 was significantly downregulated in TNBC compared to non‐TNBC subtypes (Figure 1A; Figure S1A, Supporting Information). We also analyzed mRNA levels of OVOL2 in breast cancer cell lines by quantitative reverse transcription‐PCR (RT‐qPCR). The results showed that OVOL2 mRNA levels were minimal in TNBC cell lines compared to those of non‐TNBC cell lines (Figure 1B). These results exhibit that TNBC has low expression of OVOL2.

Considering TNBC has high stemness properties and OVOL2 is critical for cell differentiation, we wondered whether OVOL2 might affect the stemness characteristics of TNBC. First, we checked the proliferation of cells by CCK8 assay. We overexpressed OVOL2 in TNBC cell lines MDA‐MB‐231, SUM159PT, and BT549 which exhibit very low OVOL2 expression, and found that OVOL2 could reduce cell proliferation (Figure 1C; Figure S1B, Supporting Information). Second, we analyzed sphere‐formation which is commonly utilized to study the stemness characteristics of cancer cells.^[^ ^21^, ^22^, ^23^ ^]^ MDA‐MB‐231 cells with OVOL2 overexpression generated smaller spheres and the rate of sphere formation decreased by 74.7% (Figure 1D). Similarly, overexpression of OVOL2 almost blocked sphere‐formation in SUM159PT and BT549 cells (Figure 1D). In addition, we assayed the in vivo tumor‐initiating capacity (TIC) of limiting dilutions of MDA‐MB‐231 cells with or without OVOL2 overexpression. With 10^5^ or 10^4^ cells injection, 100% of animals developed tumors in control groups while only 33% of animals formed tumors in OVOL2 expressed groups. Following the injection of 10^3^ cells, tumor formation occurred in 67% of cases in the control group and no tumors were observed in OVOL2‐expressed groups. (Figure 1E). Consistently, knocked down OVOL2 in OVOL2‐high luminal cell lines MCF7 and T47D raised cell proliferation and sphere formation (Figure S1C–E, Supporting Information). Additionally, OVOL2 expression was found to be significantly downregulated in sphere‐formed cells compared to adherent cells (Figure S1F, Supporting Information). Similar results were observed in the Gene Expression Omnibus (GEO) database (accession numbers GSE182532)^[^ ^24^ ^]^ (Figure S1G, Supporting Information). These results highlight the crucial role of OVOL2 in diminishing breast cancer stemness characteristics.

### 2.2 Absence of OVOL2 Enhances Fatty Oxidation and Sensitizes TNBC Cells to Etomoxir

**Figure 2:**
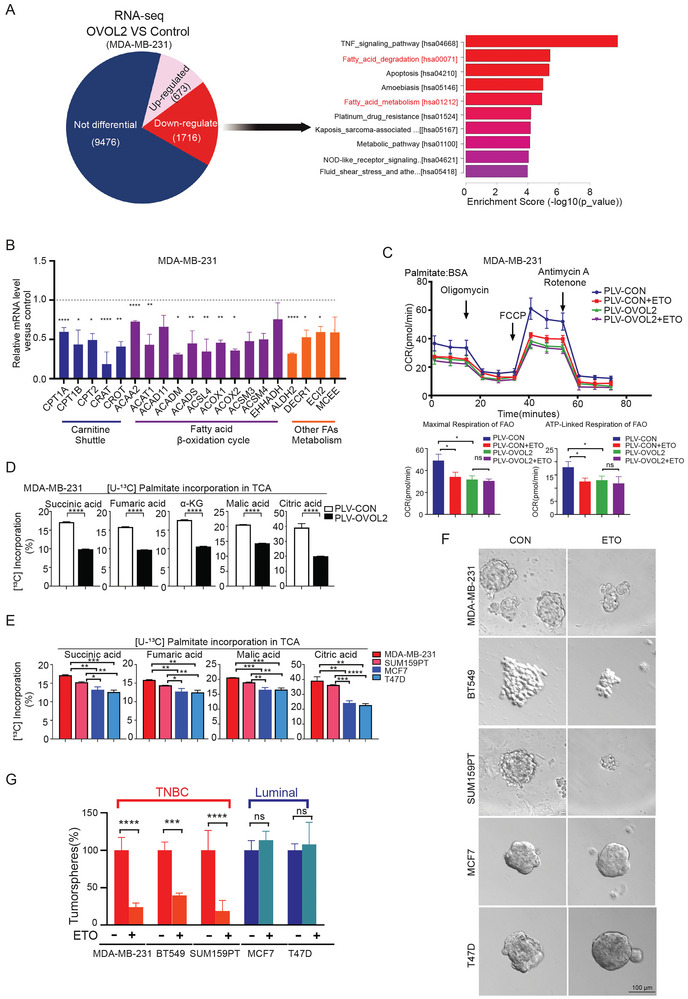
Absence of OVOL2 enhances fatty oxidation and sensitizes TNBC cells to Etomoxir. A) The left panel shows pie charts depicting the genes identified through total RNA‐seq. Upregulated and downregulated genes with a fold change of ≥1.5 are highlighted. The right panel displays the KEGG pathway enrichment analysis of the downregulated genes. B) RT–qPCR array analysis of lipid metabolism‐related genes in MDA‐MB‐231 cells. C) FAO was evaluated by measuring the FAO‐OCR using palmitate‐BSA as a substrate. The lower panel shows the ATP‐linked FAO‐OCR and maximal FAO‐OCR. D,E) U‐^13^C_16_ palmitate incorporation was evaluated by GCMS. F,G) Measurement of mammosphere formation following treatment with 50 µm ETO in different cell lines. The graphs show the sphere numbers compared to those in the control group. The TNBC subtype cells appeared to be more vulnerable to the FAO inhibitor drug. *p*‐value determined by Student's *t*‐test. The data are presented as means ± SD. The symbols *, **, *** and represent *p* < 0.05, *p* < 0.01 and *p* < 0.001, respectively.

To further explore the role of OVOL2 in TNBC cells, we performed RNA sequencing (RNA‐Seq) of MDA‐MB‐231 cells with or without OVOL2 expression and analyzed the data. The 1716 genes exhibited downregulation in OVOL2‐expressing cells. Notably, these downregulated genes included a substantial enrichment of genes associated with fatty acid degradation and metabolism. (**Figure** **2**A). We next performed RT‐qPCR analysis and observed a significant reduction in the relative mRNA levels of genes associated with fatty acid oxidation with overexpression of OVOL2 (Figure 2B). To evaluate cell FAO levels, Seahorse Bioscience's FAO assays, which utilize palmitate‐BSA as substrates, were performed to measure FAO‐specific oxygen consumption rate (FAO‐OCR) in control and OVOL2 overexpressed MDA‐MB‐231 cells. Etomoxir (ETO), an inhibitor of FAO, was used to block FAO‐OCR. As Figure 2C shows, OVOL2 overexpression significantly suppressed FAO‐OCR. Furthermore, we employed U‐^13^C‐palmitate labeling to evaluate the contribution of fatty acids to TCA cycle metabolites. OVOL2 overexpression led to a significant reduction of palmitate incorporation into TCA cycle in TNBC cells (Figure 2D; Figure S2A, Supporting Information). The uptake of U‐^13^C_16_ palmitate showed no significant difference (Figure S2B, Supporting Information). Considering OVOL2 is highly expressed in luminal cells, we knocked down it and found an increase of FAO‐OCR and utilization of palmitate in TCA cycle (Figure S2C,D, Supporting Information). Furthermore, we compared the incorporation ratio of palmitate to TCA cycle in TNBC and luminal cell lines. The results showed that MDA‐MB‐231, SUM159PT, and BT549 TNBC cell lines which exhibited lower OVOL2 expression displayed a higher incorporation ratio compared to OVOL2‐high expression MCF7 and T47D cells (Figure 2E). To gain insight into the significance of FAO in stem cell characteristics of breast cancer, we proceeded to examine sphere formation in both TNBC cell lines and luminal cell lines, with or without the administration of the FAO inhibitor ETO. The results showed that ETO significantly inhibited sphere formation in TNBC cell lines but had no impact in luminal cell lines (Figure 2F,G). This suggests that TNBC cell lines with the absence of OVOL2 exhibit a greater dependence on FAO for the promotion of sphere formation, and in OVOL2‐knockdown luminal cells, ETO could exert a blocking effect on sphere formation and cell proliferation (Figure S2E,F, Supporting Information). Taken together, these findings indicate that the absence of OVOL2 enhances FAO activity, thereby contributing to stem cell properties.

### 2.3 OVOL2 Reduces Energy Production and Antioxidant Capabilities of Cells

**Figure 3:**
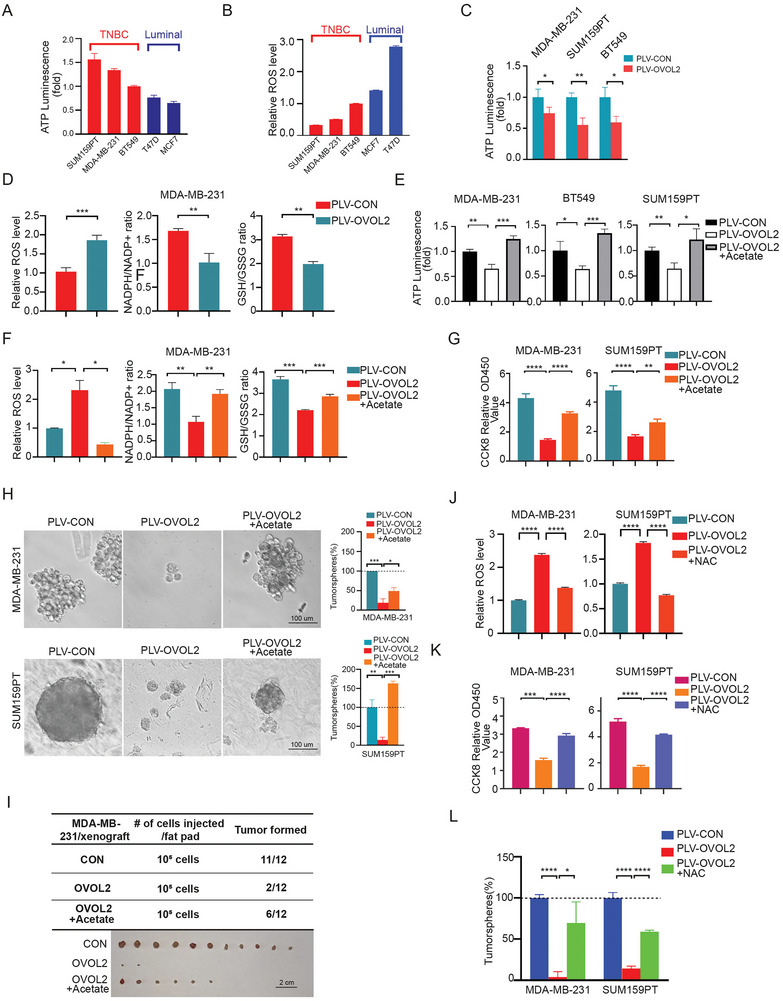
**OVOL2 suppresses cellular energy production and antioxidant capacities**. A,B) Measurement of cellular ATP levels and ROS levels in five breast cancer cell lines. TNBC subtype cells exhibit higher ATP levels and lower ROS Levels compared to Luminal subtype cells. C) The cellular ATP levels in cells were measured using an ATP assay kit. D) Overexpression of OVOL2 in MDA‐MB‐231 cells increased the ROS level and decreased NADPH/NADP+ and GSH/GSSG ratios. E) Supplementation with 5 mm acetate for 48 h compromised the OVOL2‐induced inhibition of ATP levels. F) Supplementation with 5 mm acetate for 48 h compromised the OVOL2‐induced elevation of ROS levels and reductions in the NADPH/NADP+ and GSH/GSSG ratios. G) Cell proliferation was assessed using a CCK8 assay. H) The inhibition of mammosphere formation induced by OVOL2 was compromised upon supplementation with acetate. I) Supplementation with acetate impeded the decline in tumorigenicity induced by OVOL2. J) The OVOL2‐induced intracellular accumulation of ROS was effectively quenched in the presence of NAC (2 mm). K) Evaluation of the proliferative capacity of the indicated cells via a CCK8 assay. L) NAC significantly blocked OVOL2‐induced mammosphere formation properties in breast cancer cells. *p*‐value determined by a two‐way ANOVA test. The data are presented as means ± SD. The symbols *, **, *** and represent *p *< 0.05, *p *< 0.01 and *p *< 0.001, respectively.

Incorporation of fatty acid to TCA and followed oxidative phosphorylation could generate the amount of ATP as well as NADPH which contributes to the antioxidant capabilities of cells. Consistently, higher levels of ATP and lower relative reactive oxygen species (ROS) levels were observed in TNBC compared to that of luminal cells (**Figure** **3**A,B). Further, overexpression of OVOL2 in TNBC cells led to a reduction in APT levels, NADPH/NADP^+^ ratio, and GSH/GSSG ratio (Figure 3C,D). Conversely, knocking down of OVOL2 exhibited contrary effects on these parameters in luminal cells and ETO treatment could eliminate these effects (Figure S3A,B, Supporting Information).

We next used FAO product analog acetate, the precursor of acetyl‐CoA, to treat TNBC cells with OVOL2 overexpression. The results showed that acetate could restore ATP generation and ROS levels (Figure 3E,F). Consequently, it can enhance cell proliferation, sphere formation, and tumor initiation in OVOL2‐overexpressed TNBC cells (Figure 3G–I). Moreover, to investigate the reduction of ROS levels could restore the diminished stem cell properties caused by OVOL2, we employed N‐acetylcysteine (NAC) as a scavenger of ROS scavenge. NAC treatment blocked ROS increase thereby facilitating cell proliferation and mammosphere‐forming (Figure 3J–L). These results indicated that OVOL2 abrogates FAO‐mediated energy and NADPH supply which support stem cell characteristics.

### 2.4 OVOL2 Inhibits Fatty Acid Oxidation and Stemness Characteristics by Downregulating STAT3 Signaling Pathway

**Figure 4:**
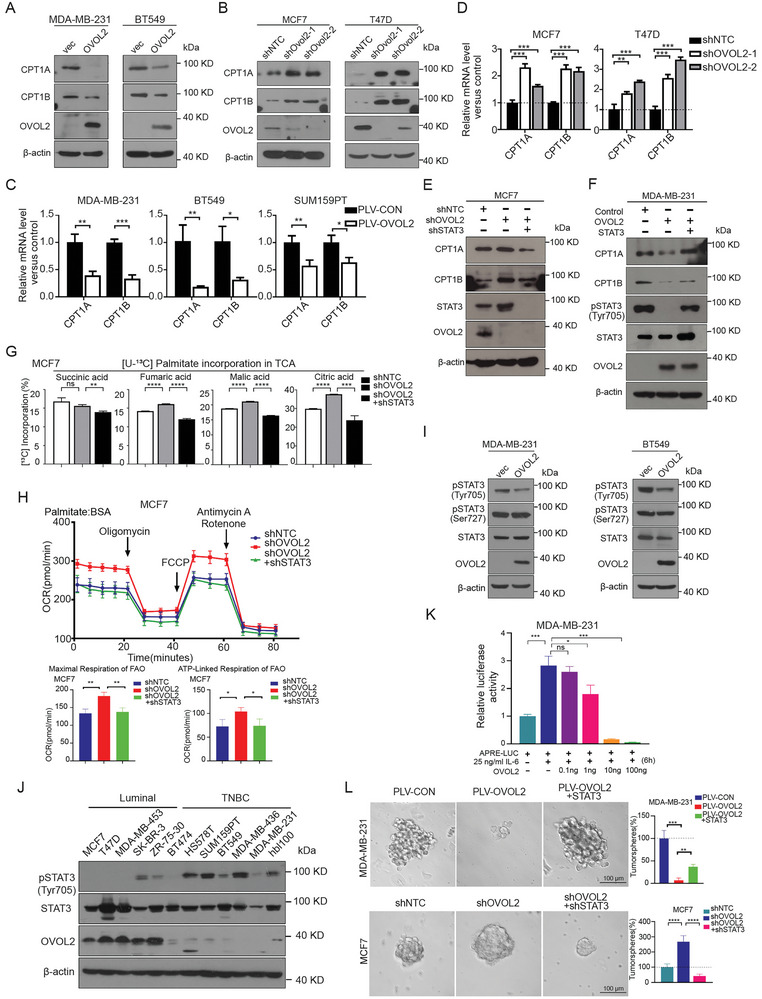
**OVOL2 inhibits fatty acid oxidation and stemness characteristics by downregulating STAT3 signaling pathway**. A) Western blot analysis of CPT1A and CPT1B in cell lines stably transduced with control or OVOL2‐encoding vectors. B) Western blot analysis of CPT1A and CPT1B expression in MCF7 and T47D cells stably transduced with control or OVOL2 shRNA. C,D) The RNA expression levels of CPT1A and CPT1B in OVOL2‐overexpressing C) and OVOL2‐knockdown D) clones were determined by RT–qPCR. E,F) Knockdown of OVOL2 was followed by the knockdown of STAT3 in MCF7 cells E). Similarly, the overexpression of OVOL2 was carried out, followed by the overexpression of STAT3 in MDA‐MB‐231 cells F). The expression levels of CPT1A and CPT1B were determined through Western blot experiments. G) U‐^13^C_16_ palmitate incorporation assay in MCF7 cells showed that STAT3 knockdown can counteract the effect of OVOL2 knockdown. H) STAT3 knockdown attenuated the effect of OVOL2 knockdown on FAO‐OCR abilities in MCF7 cells. I) Western blot analysis of OVOL2, pSTAT3 (Tyr705), and pSTAT3 (Ser705) in cells stably transduced with control or OVOL2‐encoding vectors. J) Expression of OVOL2 and phosphorylated STAT3 (pY705) in 12 human breast cancer cell lines determined by Western blotting. K) APRE‐luciferase reporter assay performed to detect the transcriptional activity of STAT3. L) STAT3 compromised the changes in mammosphere formation induced by OVOL2. The graphs show the sphere numbers compared to those in the control group. *p*‐value determined by a two‐way ANOVA test. The data are presented as means ± SD. The symbols *, **, *** and represent *p *< 0.05, *p *< 0.01 and *p *< 0.001, respectively.

We next wondered how OVOL2 inhibits fatty acid oxidation. As CPT1A and CPT1B are the rate‐limiting enzymes of fatty acid oxidation and play a critical role in maintaining the stemness characteristics of breast cancer,^[^ ^14^, ^25^ ^]^ we first checked their levels. Both protein and mRNA levels of CPT1A and CPT1B were significantly decreased in OVOL2‐overexpressing TNBC cells and elevated in OVOL2 knockdown luminal cells. (**Figure** **4**A–D). In addition, similar to OVOL2‐overexpression, knockdown of CPT1A inhibited tumor initiation in MDA‐MB‐231 xenografts in nude mice (Figure S4A, Supporting Information). These indicate that loss of OVOL2 promotes fatty acid oxidation that supports breast cancer stemness properties might though upregulating CPT1 expression.

Given the transcriptional repressor function of OVOL2, we hypothesized that it might directly modulate the transcription of CPT1. However, chromatin immunoprecipitation (ChIP) qPCR results showed OVOL2 did not bind to predicted binding sites of CPT1A and CPT1B promoters (Figure S4B, Supporting Information). It prompts us that OVOL2 might downregulate CPT1 expression via other transcription factors.

JAK/STAT3 signaling has been reported to activate FAO through the transcription of CPT1B in breast cancers.^[^ ^26^ ^]^ We observed that knockdown of STAT3 in MDA‐MB‐231 cells indeed reduced relative mRNA levels and protein levels of CPT1A and CPT1B (Figure S4C,D, Supporting Information). ChIP‐qPCR assays showed that STAT3 could directly bind to the promoter regions of CPT1A and CPT1B (Figure S4E, Supporting Information). To address whether OVOL2 inhibits CPT1 expression via STAT3, we double knocked down STAT3 and OVOL2 in MCF7 and found that loss of STAT3 suppressed shOVOL2‐induced enhancement of CPT1 expressions (Figure 4E). Overexpression of STAT3 could rescue the CPT1 expression in MDA‐MB‐231 cells with OVOL2 overexpression (Figure 4F). Moreover, the knockdown of STAT3 attenuated the increase in palmitate incorporation into TCA cycle and FAO‐mediated O_2_ consumption in OVOL2‐knockdown MCF7 cells (Figure 4G,H). These data confirm that OVOL2 suppresses CPT1 expression and FAO via STAT3.

As phosphorylation of STAT3 at tyrosine (Tyr) 705 is crucial for STAT3 transcription activity, we asked if OVOL2 affects its phosphorylation and transcriptional activity. We found OVOL2 suppressed phosphorylation of STAT3 in TNBC cells (Figure 4I). Conversely, the knockdown of OVOL2 in MCF7 cells resulted in increased STAT3 phosphorylation (Figure S4F, Supporting Information). Further, high levels of STAT3 T705 phosphorylation were observed in TNBC cells, while luminal cells exhibit comparatively lower levels. The results showed an inverse correlation between STAT3 phosphorylation and OVOL2 expression (Figure 4J). Additionally, in a STAT3‐dependent APRE‐luciferase reporter assay, OVOL2 dose‐dependently repressed IL‐6‐mediated STAT3 transcriptional activity (Figure 4K; Figure S4G,H, Supporting Information).

Moreover, the mammosphere‐forming ability of MDA‐MB‐231 cells was partially rescued by STAT3 in OVOL2 overexpression cells while knockdown of STAT3 blocked sphere formation induced by shOVOL2 in MCF7 cells. (Figure 4L). Importantly, neither activation nor silencing of STAT3 altered mRNA levels of OVOL2 (Figure S4I,J, Supporting Information). In summary, these findings indicate that OVOL2 inhibits fatty acid oxidation and stemness characteristics by suppressing STAT3 phosphorylation and activation.

### 2.5 OVOL2 Suppresses the STAT3 Signaling Pathway Through Multiple Mechanisms

**Figure 5:**
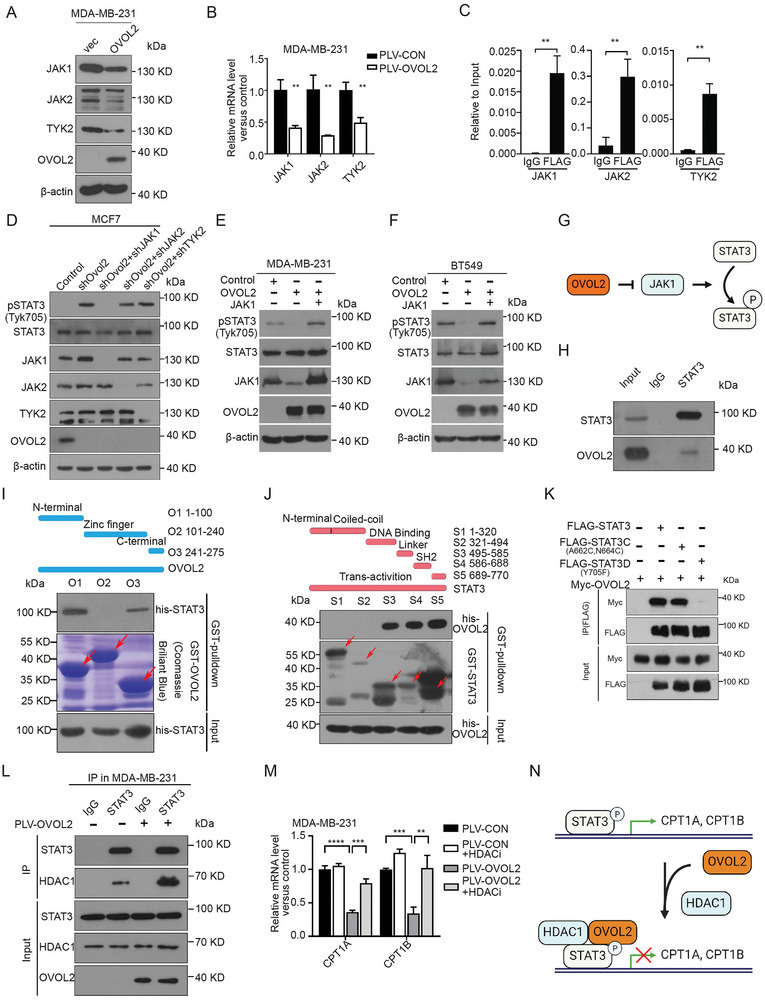
**OVOL2 suppresses the JAK/STAT3 signaling pathway through multiple mechanisms**. A,B) Western blotting and qRT‐PCR were used to measure the expression levels of JAK1, JAK2, and TYK2 upon OVOL2 overexpression in MDA‐MB‐231 cells. C) ChIP assay demonstrating the binding of OVOL2 to the promoters of JAK1, JAK2, and TYK2. The values were normalized to input DNA. D) Western blot analysis of phosphorylated STAT3 (pY705) and total STAT3 in OVOL2‐knockdown MCF7 cells with stable knockdown of JAK1, JAK2, or TYK2. E,F) Western blot showing phosphorylated STAT3 (pY705) and total STAT3 levels in OVOL2‐overexpressing cells after JAK1 overexpression. G) Schematic representation of the regulatory role of OVOL2 in inhibiting STAT3 phosphorylation through JAK1 suppression. H) In vivo interaction between endogenous OVOL2 and STAT3. Lysates of MCF7 cells were subjected to immunoprecipitation using an anti‐STAT3 antibody. I,J) Direct binding of STAT3 and OVOL2 at both the C‐terminus (aa 241–275) and N‐terminus (aa 1–100) of OVOL2 I), as well as at the linker domain (S3, aa 495–585), SH2 domain (S4, aa 586–688), and transactivation domain (S5, aa 689–770) of STAT3 J). Red arrows indicate target bands. K) Co‐IP experiments of OVOL2 and STAT3‐WT, STAT3C, and STAT3D reveal disrupted interaction between OVOL2 and unphosphorylated STAT3. Whole‐cell lysates were used for IP with an anti‐Flag antibody. L) Endogenous co‐IP experiments between HDAC1 and STAT3 in MDA‐MB‐231 cells with or without OVOL2 overexpression. M) The inhibitory effect of OVOL2 on expression levels of CPT1A and CPT1B was reversed by treatment with 2 µm tacedinaline, an HDAC1 inhibitor. (N) Schematic representation of how OVOL2 facilitates the recruitment of HDAC1 to STAT3 at CPT1A and CPT1B promoter. *p*‐value determined by Student's *t*‐test. The data are presented as means ± SD. The symbols *, **, *** and represent *p *< 0.05, *p *< 0.01 and *p *< 0.001, respectively.

JAK proteins phosphorylate STAT3 at Tyr705, initiating its transcriptional activity. We observed that OVOL2 overexpression reduced the mRNA and protein levels of three JAK family members JAK1, JAK2, and TYK2 (**Figure** **5**A,B; Figure S5A,B, Supporting Information). Moreover, ChIP qPCR results showed that OVOL 2 directly binds to their promoters (Figure 5C) and found that JAK1 was the most significant contributor to STAT3 activation induced by OVOL2 knockdown (Figure 5D; Figure S5C, Supporting Information). Consistently, JAK1 effectively restored STAT3 phosphorylation in OVOL2‐overexpressing cells (Figure 5E,F). These results indicate that OVOL2 downregulates STAT3 phosphorylation by suppressing transcription of JAK1 (Figure 5G).

We further explored whether OVOL2 interacts with STAT3. Co‐immunoprecipitation (Co‐IP) confirmed the physical interaction between OVOL2 and STAT3 (Figure 5H; Figure S5D, Supporting Information). Moreover, an in vitro pulldown assay demonstrated the direct interaction between OVOL2 and STAT3 (Figure S5E, Supporting Information). Deletion mapping showed both the C‐terminus (aa 241–275) and N‐terminus (aa 1–100) of OVOL2 were crucial for direct binding to STAT3 (Figure 5I). The linker domain (S3, aa 495–585), SH2 domain (S4, aa 586–688), and a transactivation domain (S5, aa 689–770) of STAT3 could bind to OVOL2 (Figure 5J). We next investigated whether activation of STAT3 affects its binding to OVOL2, we used constitutively active STAT3 (STAT3C, A662C N664C) and dominant negative STAT3 (STAT3D, Y705F) plasmids^[^ ^27^ ^]^ (Figure S5F, Supporting Information). Unlike STAT3 WT and STAT3C, STAT3D, which lacks activity, failed to exhibit any interactions with OVOL2 (Figure 5K).

We subsequently investigated how the binding of OVOL2 regulates the transcriptional activity of STAT3. Given that OVOL2 has been shown to recruit histone deacetylases HDAC1,^[^ ^19^ ^]^ we hypothesized that the binding of OVOL2 may facilitate the recruitment of HDAC1 to STAT3, thereby suppressing its transcriptional activity. First, we performed Co‐IP assay in MDA‐MB‐231 cells and the results indicated that STAT3 pulled down more HDAC1 in the presence of OVOL2 (Figure 5L). OVOL2 facilitates the interaction between HDAC1 and STAT3 when STAT3 is phosphorylated and activated (Figure S5G, Supporting Information). To assess the impact of OVOL2 on the association between HDAC1 and STAT3 target chromatin at the promoters of CPT1A or CPT1B, a DNA affinity precipitation assay (DAPA) was performed. The results showed that overexpression of OVOL2 enhanced the association between HDAC1 and STAT3 binding elements at the CPT1A or CPT1B promoters (Figure S5H, Supporting Information). Further, ChIP Re‐IP assays revealed that OVOL2 overexpression enhanced the association between HDAC1 and the promoters of CPT1A or CPT1B that were immunoprecipitated by anti‐STAT3 antibodies in MDA‐MB231 cells (Figure S5I,J, Supporting Information). Additionally, treatment with tacedinaline, an inhibitor of HDAC1, reversed the inhibitory effect of OVOL2 on expression levels of CPT1A and CPT1B (Figure 5M). Taken together, these results indicate that OVOL2 could bind to phosphorylated STAT3, facilitate the recruitment of HDAC1, and ultimately disrupt the transcriptional activation of STAT3 downstream genes like CPT1A and CPT1B (Figure 5N).

### 2.6 Loss of OVOL2 Promotes Breast Tumor Initiation via JAK/STAT3/CPT1 Regulated FAO in MMTV‐PyVT Mice

**Figure 6:**
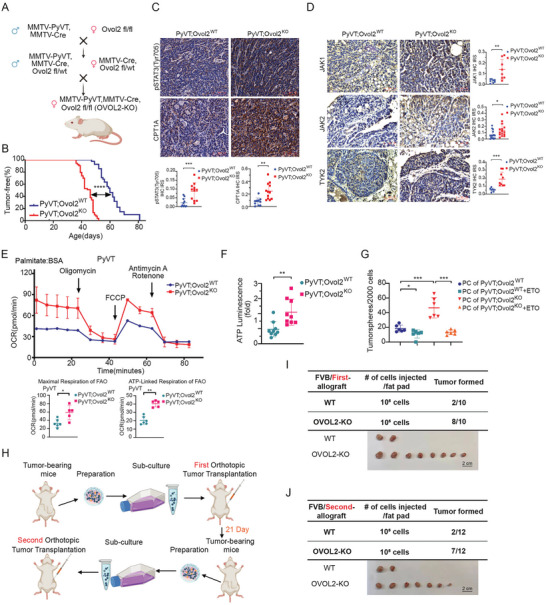
**Loss of OVOL2 promotes breast tumor initiation via JAK/STAT3/CPT1 regulated FAO in MMTV‐PyVT mice**. A) Breeding scheme illustration. B) Kaplan‐Meier plots showing mammary tumor‐free survival in female PyVT‐OVOL2^WT^ (*n* = 41) and PyVT‐OVOL2^KO^ (*n* = 54) mice. C,D) IHC staining of pSTAT3 and CPT1A C), as well as JAK1, JAK2, and TYK2 D) in mammary tumors from female PyVT and PyVT‐OVOL2^KO^ mice. The immunoreactive scores (IRSs) were calculated to analyze the IHC images. Representative images are shown, and the scale bars indicate 50 µm. E) Increased FAO‐OCR in PyVT‐OVOL2^KO^ mouse tumor cells (*n* = 5–6) indicating loss of OVOL2 function. FAO‐OCR measurements were conducted in primary cultured tumor cells isolated from PyVT mammary tumors after the second passage. F) Elevated ATP levels in PyVT‐OVOL2^KO^ mouse tumor cells (*n* = 9) due to loss of OVOL2. G) Mammosphere formation assay performed on primary cells isolated from PyVT mammary tumors after the second passage and treated with ETO. H) Schematic representation of the procedures for the first and second orthotopic injections. I,J) Enhanced tumor‐initiating ability was observed in PyVT‐OVOL2^KO^ mouse tumor cells during the first and second orthotopic injections (*n* = 10–12 mice per group) compared to normal tumor cells. The data are presented as means ± SEM. Statistical significance is represented as ** (*p* < 0.01), *** (*p* < 0.001), **** (*p* < 0.0001), ns (not significant), determined by the Gehan‐Breslow‐Wilcoxon test.

To further explore the physiological role of OVOL2 in breast cancer, we generated MMTV‐PyVT, MMTV‐Cre Ovol2 ^f/f^ mice (PyVT‐Ovol2^KO^) (**Figure** **6**A; Figure S6A, Supporting Information). We found that OVOL2 knockout alone did not induce tumor development. However, in PyVT‐Ovol2^KO^ mice, mammary gland tumors appeared earlier, indicating accelerated tumorigenesis onset (Figure 6B). We also verified that JAK/STAT3/CPT1A pathways were upregulated in Ovol2 knockout mouse tumors. Western blot analysis confirmed reduced STAT3 phosphorylation at Tyr705 in PyVT‐Ovol2^KO^ tumors compared to PyVT‐WT tumors (Figure S6B, Supporting Information). Furthermore, IHC staining revealed increased STAT3 phosphorylation and elevated expression of CPT1A, JAK1, JAK2, and TYK2 in Ovol2^KO^ tumors compared to control tumors (Figure 6C,D), suggesting that OVOL2 knockdown activates the JAK/STAT3/CPT1A pathways. We also checked FAO levels in primary breast tumor cells. The 21 days after the tumors became palpable, we euthanized the mice and isolated tumor cells to examine their metabolic characteristics. Ovol2^KO^ tumor cells showed elevated FAO‐OCRs and ATP levels (Figure 6E,F), indicating significantly enhanced FAO in the absence of OVOL2. Mammo sphere‐forming abilities were also remarkably stronger in Ovol2^KO^ tumor cells, which could be suppressed by ETO treatment (Figure 6G). Moreover, to further evaluate tumor‐initiating capacity, Ovol2^WT^, and Ovol2^KO^ tumor cells were isolated and transplanted into the mammary fat pads of recipient mice twice. We found that Ovol2^KO^ cells showed robust tumor formation, while Ovol2^WT^ cells exhibited diminished tumor‐initiating capacity (Figure 6H,I). Secondary transplantation confirmed the elevated tumor‐initiating capacity of Ovol2^KO^ cells (Figure 6J). These findings demonstrate that loss of OVOL2 facilitates mammary gland tumor initiation via JAK/STAT3/CPT1 pathway‐regulated FAO in the murine model.

Besides, we observed that primary PyVT‐Ovol2^KO^ tumors exhibited faster growth in comparison to PyVT‐Ovol2^WT^ tumors (Figure S6C, Supporting Information). Moreover, Ki67 staining indicated an increase in the proliferation of PyVT‐Ovol2^KO^ tumors (Figure S6D, Supporting Information). Additionally, OVOL2 deletion significantly reduced the median survival time and life expectancy of tumor‐bearing PyVT‐Ovol2^KO^ mice (Figure S6E, Supporting Information). The absence of OVOL2 resulted in a substantial increase in the number and size of metastatic nodules in the lungs (Figure S6F,G, Supporting Information). FACS analysis further revealed an abundance of EPCAM‐low cells in the PyVT‐Ovol2^KO^ tumor population, which is rare in PyVT‐Ovol2^WT^ tumors (Figure S6H,I, Supporting Information). The body weights of the mice indicated no significant differences between the experimental groups (Figure S6J, Supporting Information). Overall, these findings establish the suppressive role of OVOL2 in breast cancer progression, and its loss leads to an increase in the malignancy of breast tumors.

### 2.7 Targeting FAO or JAK to Inhibit Breast Tumor Progress in OVOL2 Knockout Mice

**Figure 7:**
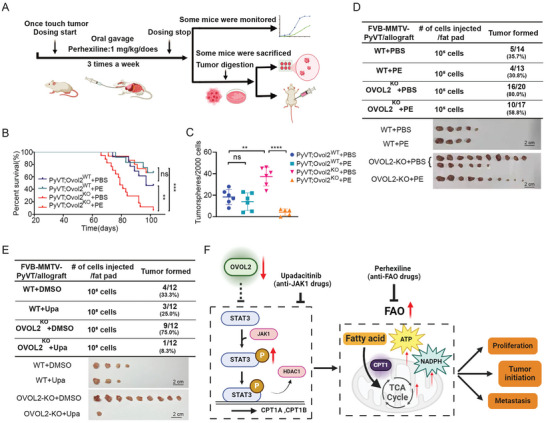
**Targeting JAK or FAO to inhibit breast tumor progress in OVOL2 knockout mice**. A) Experimental schematic for PyVT model mice treated with or without perhexiline (PE, 1 mg kg^−1^dose by oral gavage three times weekly for three weeks) and subsequent applications in different experiments. B) Survival curves of PyVT and PyVT‐OVOL2^KO^ mice treated with or without PE as described. C) A mammosphere formation assay performed on primary cells isolated from PyVT or PyVT‐OVOL2^KO^ mice treated with PBS or PE (*n* = 5–6). D,E) Injection of 1 × 10^5^ PyVT or PyVT‐OVOL2^KO^ tumor cells from PyVT model mice treated with or without perhexiline D) or upadacitinib E) into the mammary fat pads of FVB mice. After 21 days, mice were sacrificed, and tumors were collected. F) Schematic illustrating the effect of OVOL2 on breast cancer cells. The data are presented as means ± SEMs. Statistical significance is represented as ** (*p* < 0.01), *** (*p* < 0.001), **** (*p* < 0.0001), ns (not significant), determined by the Gehan–Breslow–Wilcoxon test.

Because ETO treatment could reduce the sphere formation of tumor cells in the above experiments, we deduced that inhibition of FAO might be an ideal strategy for the prevention of tumor initiation. Perhexiline, a CPT1 inhibitor used clinically for antianginal treatment,^[^ ^28^ ^]^ was administrated to PyVT‐Ovol2^WT^ and PyVT‐Ovol2^KO^ mice after the tumors were touchable (**Figure** **7**A). After continuous treatment for 21 days, some mice were kept to monitor survival and the others were sacrificed to isolate tumor cells which were subjected to mammosphere‐forming assays as well as orthotopic transplantation (Figure 7A). Overall survival was significantly improved by perhexiline treatment in OVOL2‐deficient mice compared to WT mice (Figure 7B). Moreover, the sphere formation of Ovol2^KO^ tumor cells was reduced by FAO inhibition in vivo with perhexiline treatment via gavage (Figure 7C). Tumor cells lacking OVOL2 exhibited higher tumorigenic capability, which was effectively inhibited by perhexiline treatment (Figure 7D). Our results demonstrated that OVOL2 deletion promotes tumor initiation in MMTV‐PyVT mice via the regulation of FAO, thus implicating the potential clinical value of FAO inhibitors for breast cancer treatment.

Furthermore, we also observed the effects of perhexiline on the development of primary mouse breast tumors. PyVT‐Ovol2^KO^ tumors with Perhexiline treatment exhibited significantly decreased tumor growth compared to the PBS‐treated groups (Figure S7A,B, Supporting Information). Imaging and quantification of metastases showed a remarkable reduction in lung metastatic spread in PyVT‐Ovol2^KO^ mice treated with perhexiline (Figure S7C–E, Supporting Information). IHC staining for the proliferation marker Ki67, the epithelial marker ECAD, and the EMT marker Vimentin revealed that perhexiline inhibited the increased cell proliferation and transformation observed in OVOL2‐deficient tumors compared to control tumors (Figure S7F, Supporting Information).

Similarly, in vivo, we aimed to validate the JAK/STAT pathway as a critical downstream target of OVOL2. The administration of upadacitinib (Upa), a JAK1 inhibitor, substantially attenuated the tumorigenic promotion induced by OVOL2 deficiency (Figure 7E).

These findings clearly indicate the requirement of FAO and JAK/STAT3 pathway for breast tumor progression in mice with OVOL2 deficiency. Moreover, these results provide compelling evidence that loss of OVOL2 activates JAK/STAT3 pathway and enhances FAO which makes tumors more susceptible to perhexiline or upadacitinib treatment (Figure 7F). This suggests that targeting FAO or JAK/STAT pathway represents a promising therapeutic strategy for OVOL2‐deficient TNBC.

## 3 Discussion

Triple‐negative breast cancer is the most aggressive subtype of breast cancer. The lack of ER, PR, and HER2 receptors makes it insensitive to hormone‐based or receptor‐targeted therapies. Thus, there is a pressing need to discover new characteristics of TNBC and devise novel and effective therapeutic approaches to combat this challenging disease. In this study, we found that OVOL2 expression was significantly downregulated in TNBC compared to other subtypes. Overexpression of OVOL2 in TNBC cell lines reduced breast cancer stemness characteristics including cell proliferation and sphere formation both in vitro and in vivo. Understanding how OVOL2 inhibits the development of TNBC progressing may provide crucial clues for the development of new therapeutic treatments.

Metabolic remolding is a hallmark of cancer. Traditionally, it has been believed that tumor cells predominantly rely on anaerobic glycolysis, a phenomenon known as the “Warburg effect,” even in the presence of oxygen. Hypoxia could induce downregulation of OVOL2 thereby increasing glycolysis.^[^ ^29^ ^]^ However, it is important to note that cancer cells exhibit metabolic adaptability, which depends on the availability of nutrients and oxygen.^[^ ^30^ ^]^ This metabolic flexibility enables cells in diverse nutritional and oxygen environments to establish metabolic coupling, thereby promoting cancer cell proliferation and tumor growth. Substantial evidence demonstrates the significance of fatty acid oxidation in breast tumor progression.^[^ ^31^, ^32^, ^33^ ^]^ Compared to glucose, fatty acid could generate more energy and antioxidants through fatty acid oxidation.^[^ ^5^, ^34^, ^35^, ^36^ ^]^ Here, by RNA‐Seq analysis, we discovered that OVOL2 negatively regulated fatty acid degradation in TNBC cells. OVOL2 loss in TNBC resulted in elevated fatty acid oxidation. OVOL2 overexpression significantly inhibited palmitate‐mediated O_2_ consumption and the incorporation of ^13^C‐palmitate into TCA cycle thereby reducing ATP levels and upregulating ROS levels. The supplementation of acetate as a direct product of fatty acid oxidation restored the effect elicited by OVOL2 expression. These indicate OVOL2 functions as a negative regulator in FAO‐mediated metabolic remodeling and antioxidant defense.

CSCs possess the ability of self‐renewal, and tumor initiation, and are associated with tumor growth, metastasis, drug resistance, and recurrence.^[^ ^37^ ^]^ Recent studies have highlighted the crucial role of FAO in cancer stem cells.^[^ ^38^, ^39^ ^]^ Inhibiting FAO has been found to increase the sensitivity of breast cancer CSCs to chemotherapy drugs.^[^ ^6^ ^]^ For instance, in colorectal cancer, FAO‐generated acetyl‐CoA is transferred by P300 to promote H3K27 acetylation of the Nanog promoter, thereby enhancing Nanog expression and inducing cellular dormancy akin to quiescent cancer stem cells.^[^ ^40^ ^]^ FAO utilization also circumvents the need for amino acid metabolism, consequently conferring resistance to drugs like venetoclax or azacitidine in acute myeloid leukemia stem cells.^[^ ^41^ ^]^ To evaluate the stemness features of CSCs, we employed the sphere‐forming assay and in vivo tumor transplantation assay as indicators. The sphere‐forming assay creates a relatively deprived environment for cells and assesses their stemness based on their resistance to anoikis, a form of programmed cell death induced by detachment from the extracellular matrix.^[^ ^42^ ^]^ On the other hand, the in vivo tumor transplantation assay directly evaluates the tumor‐initiating capability of cancer cells, serving as a measure of their stemness.^[^ ^43^ ^]^ Through these assessments, we made an intriguing observation that OVOL2 not only possesses the ability to inhibit sphere formation and tumor‐initiating capacity in cells cultured in vitro but also exerts a significant impact on the stemness characteristics of breast tumor cells in the PyVT mouse model. Specifically, the secondary transplantation experiment revealed that the loss of OVOL2 in mouse breast tumor cells resulted in enhanced and more stable tumor‐initiating capacity, further highlighting the crucial role of OVOL2 in regulating CSC properties in vivo. Additionally, we observed that impaired FAO contributed to the reduction in stemness characteristics in OVOL2‐overexpressing cells and acetate could restore the sphere‐forming abilities. In a mouse model, OVOL2 deletion resulted in enhanced FAO capability, increased tumor‐initiating capacity, and accelerated tumor growth.

Notably, the absence of OVOL2 makes TNBC and primary breast tumors become more reliant on fatty acid oxidation to sustain rapid growth and tumor progression.

FAO inhibitor ETO could effectively block sphere formation in OVOL2 absent TNBC cells but not in OVOL2‐high‐expressed luminal cells. Besides, perhexiline, a CPT1 inhibitor used clinically for antianginal treatment,^[^ ^28^ ^]^ significantly inhibited tumor initiation and promoted mice survival in OVOL2 KO mice not in OVOL2 WT mice. These findings emphasize the promising role of OVOL2 as a predictive marker for sensitivity to FAO inhibitors in breast cancer. This discovery lays the foundation for the future precision management of breast cancer.

We discovered that OVOL2 downregulates FAO via JAK1‐STAT3 pathway. STAT3 has emerged as a key regulator of lipid metabolism. In breast cancer, prior studies have demonstrated that STAT3 directly binds to the promoter of CPT1B, promoting its transcriptional regulation, resulting in an enhanced FAO.^[^ ^14^ ^]^ Consistently, we have demonstrated that STAT3 binds to the promoters of both CPT1A and CPT1B, and depletion of STAT3 leads to a decrease in the expression levels of CPT1A and CPT1B (Figure S4C–E, Supporting Information). Furthermore, multiple lines of evidence in our study have demonstrated that the overexpression of OVOL2 impedes the expression of CPT1A and CPT1B, involved in FAO, by suppressing the signaling of STAT3. The phosphorylation status of STAT3 elevated in TNBC compared to that in other non‐TNBC subtypes. We discovered that OVOL2 exerts inhibitory effects on STAT3 by directly repressing the expression of JAK1, thereby restraining the phosphorylation of STAT3 at the Tyr705. Additionally, OVOL2 binds to phosphorylated STAT3, enhancing the recruitment of HDAC1 which is crucial for repressing the transcriptional activity of STAT3.^[^ ^44^ ^]^ Our study reveals that the STAT3/OVOL2/HDAC1 protein complex binds to the STAT3 binding site at the CPT1A and CPT1B promoters, leading to the inhibition of CPT1A and CPT1B expression.

Moreover, OVOL2 may impact lipid catabolism through multiple mechanisms. The tumor necrosis factor (TNF) signaling pathway is closely associated with lipid metabolism.^[^ ^45^ ^]^ Previous studies have reported that TNF induces lipolysis in human adipocytes by down‐regulating the lipid droplet‐associated protein perilipin (PLIN).^[^ ^46^ ^]^ Additionally, TNF inhibits the accumulation of acetyl‐CoA carboxylase, the rate‐limiting enzyme in long‐chain fatty acid biosynthesis, in pre‐adipocyte cell lines, thus reducing its activity.^[^ ^47^ ^]^ Recent reports have also demonstrated that TNF suppresses fatty acid metabolism and oxidative phosphorylation while promoting the development of fatty liver.^[^ ^48^ ^]^ Notably, our RNA‐seq analysis revealed a significant enrichment of the TNF signaling pathway. Therefore, we cannot exclude the possibility that OVOL2 may affect lipid catabolism in breast cancer cells by modulating the activity of the TNF pathway.

In summary, our study reveals a novel mechanism of OVOL2 as a tumor suppressor in breast cancer, inhibiting tumor stemness and metastasis through STAT3 signaling pathway and FAO inhibition. Modulating these molecular processes may offer new targeted therapeutic strategies for cancer.

## 4 Experimental Section

### Reagents

Carmine staining solution (Cat# G3930) was from Solarbio. B‐27 (Cat# 17504044) was from Gibco. Agarose (Cat# 2276GR025) was from BioFroxx. CCK8 (Cat# k1018) was from APExBIO. Poly (deoxyinosine‐deoxycytosine) [poly(dI‐dC)] (Cat# 10108812001) was from Roche. CellROX Oxidative Stress Reagents (Cat# C10444) and Pierce Glutathione Agarose (Cat# 16101) were from Thermo Fisher. XF DMEM (Cat# 103575‐100) was were from Agilent. U‐^13^C_16_ palmitate (Cat# CLM‐409‐0.5) was from Cambridge Isotope Laboratories. Methoxyamine (Cat# M109434) was from Aladdin. Perhexiline (Cat# HY‐B1334A), Leptin (Cat# HY‐P7232), and IL‐6 (Cat# HY‐P7044) were from Med Chem Express. Polyethylenimine Linear MW 40000 (Cat# MX2203) was from Maokangbio. Sodium acetate (Cat# S2889) was from Sigma and was dissolved in ddH2O for use in cell culture applications. Polybrene (Cat# H9268‐5G), MSTFA (Cat# M‐108‐5×1ML), MTBE (Cat# 34875), Carnitine (Cat# C0158) and palmitate (Cat# P9767) were from Sigma. Upadacitinib (Cat# S8162), Tacedinaline (Cat# S2818) and Y‐27632 2HCl (Cat# S1049) were from SELLECK. TRIzol (Cat# 15596018) was from Life Technologies. UltraSYBR Mixture (Cat# CW0957H) was from Cwbiotech. Streptavidin Magnetic Beads (Cat# P2151), RIPA buffer (Cat# P0013B), and DAPI (Cat# C1002) were from Beyotime. ECL detection system (Cat# RM00021) was from ABclonal.

### Mice and Mammary Fat Pad Xenograft Model

MMTV‐PyVT, MMTV‐Cre, OVOL2^f/+^, and FVB mice were used to generate conditional OVOL2 knockout and control mice. Experimental mice (7–14‐week‐old) were treated with perhexiline (PE, 1 mg kg^−1^ dose^−1^) or PBS by oral gavage three times weekly for 3 weeks. FVB mice and nude mice were used to establish the mammary fat pad allograft model with tumor cells from MMTV‐PyVT and MMTV‐PyVT‐OVOL2^KO^ mice. Nude mice (5‐week‐old females) were purchased from Shanghai SLAC Laboratory Animal Center. Mouse tumor cells were dissociated and isolated from mammary tumors as described in the “Primary cell preparation method” section. Single‐cell suspensions (50 µL; diluted in PBS with 50% Matrigel on ice) were injected into the fat pads of 8‐week‐old FVB mice. The secondary orthotopically injected mice were generated by isolating cells from the remaining tumors and transplanting these cells into FVB recipient mice. The tumor size was measured with calipers, and the tumor volume was determined using the following equation: volume = length× width^2^×0.5. All animals were maintained in the Laboratory Animal Center at Xiamen University (China) in accordance with institutional guidelines. All mouse protocols and experiments were approved by the Animal Ethics Committee of Xiamen University (acceptance no. XMULAC20190043).

### Primary Cell Preparation Method

Freshly harvested mammary tumors were minced into 1–2 mm pieces and were then placed in tumor digestion medium (RPMI‐1640 medium supplemented with 200 U mL collagenase Type IV, 100 U mL hyaluronidase, 25 mm HEPES, 5% fetal bovine serum (FBS), and 1% penicillin–streptomycin (PS) to a 3× volume of each tissue sample) for up to 2 h at 37 °C in a shaker at 50 rpm. The digested tissues were then transferred into a 37 °C incubator overnight. The digestion status was assessed microscopically, and cell fractionation was initiated if only small tissue pieces were observed.

The digested cell clumps were transferred into 50 mL sterile tubes and centrifuged at 40 × g for 1 min. The supernatants were transferred into new 50 mL sterile tubes and centrifuged at 200 × g for 4 min. The supernatants were discarded, and the pellets obtained were washed in cold PBS and suspended in Dulbecco's modified Eagle's medium (DMEM) containing 10% FBS and 10 µm Y‐27632 2HCl (a ROCK inhibitor, Selleck, S1049). The obtained cells were passaged and used for other experiments.

### Cell Lines and Cell Culture/Transfection

Cell lines, including MDA‐MB‐231, MDA‐MB‐453, HBL100, HS578T, SK‐BR‐3, MCF7, and HEK293T, were purchased from the American Type Culture Collection (ATCC; Rockville, MD) and maintained in DMEM (Biological Industries, cat# 01‐052‐1ACS) supplemented with 10% FBS (FBSCN500‐S, AusGeneX). Other cell lines, including BT549, BT474, ZR‐75‐30, SUM159PT, and T47D, were purchased from ATCC and cultured in RPMI‐1640 medium (Biological Industries, cat# 01‐100‐1ACS) supplemented with 10% FBS. MDA‐MB‐436 cells were purchased from ATCC and maintained in L‐15 medium (HyClone, with L‐glutamine, SH30525) supplemented with 10% FBS, 10 µg mL^−1^ insulin (Sigma–Aldrich) and GlutaMAX (Gibco, 100×, 35050–061). All cells were cultured at 37 °C in a humidified incubator with 5% CO2. Transient transfection of cells was conducted using PEI (Polyethylenimine Linear MW 40000, cat# MX2203, Maokangbio) according to the manufacturer's protocol.

### Lentiviral System Construction

The pBOBi lentiviral (pLV) vector was used to overexpress target genes, and the lentiviral vector pLKO.1 was used to express shRNAs in human breast cancer cells. For lentivirus packaging, subconfluent HEK 293T cells cultured in 10 cm culture dishes were cotransfected with the lentiviral vector (8 µg), the packaging plasmid pHR (6 µg), and the envelope plasmid pVSV‐G (2 µg) using PEI. Eight hours after transfection, the transfection medium was replaced with fresh DMEM supplemented with 10% FBS. Viral supernatants were collected and filtered through a Millex‐HV 0.45 µm filter (Merck Millipore, SLHVR33RB). For lentiviral transduction, cells were transduced with lentiviral vectors in the presence of polybrene (Sigma, H9268‐5G) for 24 h, and stable cell lines were selected with puromycin. The overexpression or knockdown efficiency of the target gene was validated by western blotting or qRT–PCR 48 h after lentiviral transduction.

The sequences of the shRNAs targeting mRNAs are listed below (5’→3’).

pLKO.1 human OVOL2 shRNA:

GCTGGGATGAGCTCCCGGATGAGAA and ACATCCGCACACCAGGAGAAT

pLKO.1 human STAT3 shRNA:

GCACCTTCCTGCTAAGATTCA and GCACTTGTAATGGCGTCTTCA

pLKO.1 human JAK1 shRNA:

GCACAGAAGACGGAGGAAATG and GGTGGAAGTGATCTTCTATCT

pLKO.1 human JAK2 shRNA:

GCAACTTGGCAAGGGTAATTT and GCTTTGTCTTTCGTGTCATTA

pLKO.1 human TYK2 shRNA:

GAGATCCACCACTTTAAGAAT and CGAGCACATCATCAAGTACAA

pLKO.1 human CPT1A shRNA:

GCCATGAAGCTCTTAGACAAA and CGATGTTACGACAGGTGGTTT

### Quantitative Reverse Transcription–Polymerase Chain Reaction (qRT–PCR)

Total RNA from breast cancer cells was extracted using TRIzol (Life Technologies, 15596018) according to the manufacturer's instructions. cDNA was synthesized with oligo(dT) primers using 5× All‐In‐One RT MasterMix (abmGood, cat# G490) according to the manufacturer's instructions. RT–qPCR was performed using UltraSYBR Mixture (Cwbiotech, cat# CW0957H) according to the manufacturer's instructions with the RT–qPCR primers listed in Table S1 (Supporting Information) in a CFX ConnectTM Real‐Time PCR Detection System (Bio‐Rad) with the following amplification conditions: 95 °C for 5 min, followed by 44 cycles at 95 °C for 10 s and 60 °C for 1 min. 18S rRNA was used as the internal control. All samples were analyzed in biological and technical triplicate. The 2−ΔΔCT method was used to calculate fold changes in relative gene expression levels.

### Western Blot Analysis

Proteins from cultured cells were extracted with RIPA buffer (Beyotime, cat# P0013B), and protein concentrations were determined using a BCA Protein Assay Kit (Pierce, Thermo #23235) according to the manufacturer's instructions. Proteins were loaded onto 8–10% SDS–PAGE gels for electrophoresis and were electrotransferred to a 0.45 µm Immobilon‐P PVDF membrane (Millipore, MA, USA). The membrane was blocked with 5% bovine serum albumin (BSA) in TBST (150 mm NaCl, 20 mm Tris HCl, and 0.05% (v/v) Tween 20) for 1 h at room temperature and was then incubated with specific primary antibodies overnight at 4 °C. Next, the membrane was incubated with peroxidase‐conjugated secondary antibodies (Pierce goat anti‐rabbit IgG or Pierce goat anti‐mouse IgG) for 1 h at room temperature, and chemiluminescence signals were detected using an ECL detection system (ABclonal, cat# RM00021). Signals were visualized by exposure to film.

Antibodies used for western blotting were as follows: anti‐STAT3 (Cat# 9139, CST), anti‐phospho‐STAT3‐Tyr705 (Cat# 9145, CST), anti‐phospho‐STAT3‐Ser727 (Cat# AP0474, ABclonal), anti‐CPT1A (Cat# A5307, ABclonal), anti‐CPT1B (Cat# A6796, ABclonal), anti‐JAK1 (Cat# 66466‐1‐Ig, Proteintech), anti‐JAK2 (Cat# 3230, CST), anti‐TYK2 (Cat# A2128, ABclonal), anti‐HDAC1 (Cat# 5356, CST), anti‐HDAC1 (Cat# ab280198, Abcam), anti‐E‐cadherin (Cat# 3195, CST), anti‐Vimentin (Cat# 5741, CST), anti‐GST (Cat# ab9085, Abcam), anti‐OVOL2 (Cat# NBP2‐42907, Novus), anti‐OVOL2 (Cat# sc‐515001, Santa Cruz), anti‐Flag‐tag (Cat# 14793S, CST), anti‐HA‐tag (Cat# H6908, Sigma), anti‐Myc‐tag (Cat# C3956, Sigma), anti‐His tag (Cat# AE003, ABclonal), anti Ki67 (Cat# GTX16667, GeneTex), anti‐β‐actin (Cat# A1978, Sigma).

### Flow Cytometry

To analyze the cellular composition of mammary tumors, digested tumor cells were prepared as described above (“Primary cell preparation method” section). In addition, the digested cell clumps were transferred into 50 mL sterile tubes and centrifuged at 450 × g for 10 min. The pellets were resuspended in a Red Blood Cell Lysis Buffer (154 mm NH_4_Cl, 10 mm KHCO_3_, and 0.1 mm EDTA; pH 8.0) with gentle pipetting and sequentially incubated with prewarmed 0.25% trypsin‐EDTA and 0.5 mg mL^−1^ DNase I (NEB, #M0303S) for 5 min and centrifuged at 450 × g for 10 min. The pellets obtained were washed in cold PBS and filtered through 100 µm cell strainers. Single‐cell suspensions were maintained in MACS buffer (PBS supplemented with 2% FBS, 1% PS, and 1 mm EDTA) and used for flow cytometric staining with fluorescently labeled antibodies.

### Immunohistochemical (IHC) and Hematoxylin and Eosin (H&E) Staining

After overnight fixation with formalin at 4 °C and paraffin embedding, mammary tumor tissues were deparaffinized with 100% xylene and a descending ethanol series for further peroxidase IHC staining using an UltraSensitiveTM SP (Mouse/Rabbit) IHC Kit (MXB Biotechnologies, KIT‐9710) and a DAB Detection Kit (MXB Biotechnologies, DAB‐2031). Paraffin or frozen tissue sections were incubated with a primary antibody diluted in antibody diluent (0.1% Triton X‐100 and 2% BSA in PBS) overnight at 4 °C. After incubation with a secondary antibody, 5‐µm‐thick sections were stained using DAB and costained with hematoxylin to visualize the staining of the protein of interest. Mouse tissue sections were stained with anti‐JAK1 (1:100), anti‐JAK2 (1:100), anti‐TYK2 (1:100), anti‐OVOL2 (1:100), anti‐phospho‐Stat3 (Tyr705) (1:100), anti‐CPT1A (1:200), anti‐ECAD (1:200), anti‐Vimentin (1:200) and anti‐Ki67 (1:200) primary antibodies. For H&E staining, tissues were fixed with 4% formaldehyde and embedded in paraffin. The 5‐µm‐thick paraffin sections were dried at 65 °C for 2 h. The tissues were deparaffinized using 100% xylene and a descending ethanol series (100%, 95%, 80%, and 70%) and were then stained with H&E (Bioengineering Institute of Jiancheng, Nanjing, China, cat# D006) according to the manufacturer's instructions.

### Oncomine Analysis and Gene Expression Omnibus (GEO) Database Analysis

The UALCAN database (ualcan.path.uab.edu) and Oncomine database (www.oncomine.org) were used to analyze differences in OVOL2 mRNA expression levels between TNBC and non‐TNBC samples based on the following thresholds: *p* value <0.0001, minimum two‐fold change in gene expression and gene rank in the top 10%.

### RNA‐Seq Analysis

Cells were collected, and total RNA was isolated using a TRIzol Kit following the manufacturer's instructions. Then, total RNA was treated with RNase‐free DNase I and enriched for poly(A) RNA using an NEBNext Poly(A) mRNA Magnetic Isolation Module (New England Biolabs). All RNA was then fragmented by adding a fragmentation buffer. Next, cDNA was synthesized, and PCR was performed using a KAPA Stranded RNA‐Seq Library Prep Kit (Illumina) to construct the final cDNA library. RNA‐seq libraries were subjected to QC analyses and were sequenced using an Agilent 2100 Bioanalyzer. A Perl program was written to select clean reads by removing low‐quality sequences and reads containing adaptor sequences. Raw sequencing data (fastq) were quality‐checked with FastQC, aligned to human genomes, and further used to calculate FPKM values using quartile normalization. Differentially expressed genes between the control and OVOL2 overexpression groups were identified using quantile normalization and the per‐condition dispersion method. Genes with FPKM>1 were retained for further analysis and were used to generate the pre‐ranked gene list. Gene set enrichment analysis (GSEA) was performed on the preranked gene list. The raw data have been submitted to the NCBI Sequence Read Archive (SRA) database, and the accession number is PRJNA848624.

### Mammosphere Formation Assay

Single‐cell suspensions of cell lines (10 000 cells mL^−1^) were cultured in 24‐well ultra‐low attachment plates (Corning, 3473) in DMEM/F12 (Invitrogen, Waltham, MA, USA) containing B‐27 (1:50, Invitrogen), 20 ng mL^−1^ epidermal growth factor (EGF; BD Biosciences, San Jose, CA, USA), 20 ng mL^−1^ basic fibroblast growth factor (bFGF; BD Biosciences), 4 µg mL^−1^ insulin (Sigma–Aldrich) and 1% methylcellulose. Mammosphere cultures were fed every 3 days, and mammospheres were counted after 2–3 weeks.

### ATP Measurement

Cellular ATP levels were measured with a luminometric method using an ENLITEN ATP assay system (Promega). In brief, cells were seeded into a 24‐well plate, and cellular ATP was extracted by incubation with 100 µL of trichloroacetic acid (2%) for 2 min followed by centrifugation at 20 000 × g at 4 °C for 3 min. The supernatants were neutralized using a neutralizer solution (20× 0.33m KOH with 0.1m Tris‐acetate; pH 7.75), and 40 µL of each supernatant was then added to the same volume of rL/L reagent for measurement of ATP‐driven chemiluminescence signals in a luminometer.

### Seahorse Assay

A Seahorse XF96 Extracellular Flux Analyzer (Seahorse Bioscience) was used to evaluate the oxygen consumption rate (OCR) and FAO capacity in breast cancer cells according to the manufacturer's instructions. All cancer cells and murine primary mammary tumor cells were obtained as described above. Cells (10 000 live cells per well) were plated in an XF96 cell culture plate at 90% confluence. For determination of the OCR, the cell culture medium was replaced with XF DMEM (Agilent, #103575‐100) supplemented with 25 mm glucose, 1 mm sodium pyruvate, and 2 mm glutamine and incubated for 1 h at 37 °C prior to the assay. Mitochondrial stress was induced with a Mito Stress Test Kit (Agilent, 103015–100) by sequential injection of 1 µm oligomycin, 1 µm FCCP, and 0.5 µm rotenone and antimycin A. For measurement of the FAO rate, the presence of palmitate‐BSA in an assay medium in combination with etomoxir (ETO) can drive cells to oxidize fatty acids (FAs) and can reveal the portion of the OCR signal generated by FAO. As described in detail below, when cells were 80–90% confluent, the culture medium was replaced with a substrate‐limited medium (XF DMEM supplemented with 0.5 mm glucose, 1.0 mm glutamine, 0.5 mm carnitine, and 1% FBS) for 4 h at 37 °C. The substrate‐limited medium was then replaced with FAO assay KHB buffer (111 mm NaCl, 4.7 mm KCl, 1.25 mm CaCl_2_, 2.0 mm MgSO_4_, 1.2 mm NaH_2_PO_4_) supplemented with 2.5 mm glucose, 0.5 mm carnitine, 5 mm HEPES and adjusted to pH 7.4 for 45 min prior to the assay. To quantify FAO, the assay medium was supplemented with 50 µm ETO 15 min before the assay. BSA control (0.17 mm BSA (Low Free Fatty Acid, MP Biomedicals, cat# 199899) and 150 mm NaCl; pH 7.2) or palmitate (Sigma, cat# P9767) conjugated to BSA (6:1) was added at a final concentration of 175 µm as exogenous fatty acids immediately before the measurement. Mitochondrial stress was induced as described above. The maximal OCR was calculated by subtracting the nonmitochondrial OCR from the OCR after FCCP treatment, while the ATP‐linked OCR was calculated by subtracting the OCR after oligomycin treatment from the basal OCR. The FAO‐associated OCR was measured according to the instructions of the Agilent Seahorse XF Palmitate‐BSA FAO Substrate Quickstart Guide. Cells were counted after completion of the experiment to normalize the rates in the individual wells, and the data were analyzed with Agilent Wave software v.2.6.

### Gas Chromatography–Mass Spectrometry (GC–MS) Analysis

For tricarboxylic acid (TCA) metabolite extraction in U‐^13^C_16_ palmitate and U‐^13^C_2_ acetate cellular labeling experiments, breast cancer cells cultured to ≈80–90% confluence were treated with 0.5 mm U‐^13^C_16_ palmitate (Cambridge Isotope Laboratories, Inc.; cat# CLM‐409‐0.5) and 1 mm carnitine (Sigma, cat# C0158) for 2 h prior to harvesting. Cells were washed three times in precooled PBS and quenched by bringing the bottom (outer wall) of the culture dish in contact with liquid nitrogen. Metabolites were extracted using ice‐cold 80% methanol (methanol/water, v/v) and transferred to a new tube using a cell scraper. Samples were centrifuged at 13 500 rpm for 10 min at 4 °C to remove cell debris. Supernatants were transferred into 1.5 mL Eppendorf tubes and dried in a vacuum centrifugal enrichment system at 4 °C (Labconco Corporation), and 50 µL of methoxyamine (Aladdin, cat# M109434) in pyridine solution (20 mg mL^−1^) was added. After vortexing for 60 s and ultrasonication for 5 min in an ice bath, the oximation reaction was conducted at 37 °C in a water bath for 1.5 h. Subsequently, 50 µL of N‐methyl‐N‐(trimethylsilyl)trifluoroacetamide (MSTFA; Sigma, cat#M‐108‐5×1 ML) was added, and the final silylation derivatization was performed at 37 °C for 1.5 h. The mixture was centrifuged at 15 000 × g for 10 min at 4 °C, and 60 µL of each sample was transferred into injection vials (CNW Technologies, P/N: VDAP‐4025BS‐629‐100, P/N: VAAP‐32009E‐1232A‐100, P/N: VEAP‐5394‐09FRB‐100) and analyzed by GC–MS within 24 h. To quantify U‐^13^C_16_ palmitate in medium supernatants, 200 µL of homogenized medium samples were extracted with 400 µL of methyl tert‐butyl ether (MTBE; Sigma, cat# 34875) and 80 µL of methanol, followed by vortexing for 30 s and ultrasonication for 10 min in an ice bath. After centrifugation at 3000 rpm for 15 min at 4 °C, 300 µL of each supernatant was transferred to a 1.5 mL Eppendorf tube, dried under nitrogen gas, and dissolved in 100 µL of MSTFA for subsequent GC–MS analysis (Agilent Technologies; Agilent 5977B).

### Immunoprecipitation (IP)

Cells were lysed in lysis buffer (150 mm NaCl, 20 mm Tris‐HCl, 1 mm EDTA, 1 mm EGTA, and 1% Triton X‐100) supplemented with 1 mm PMSF. The cell lysates were precleared with protein A/G beads and were then immunoprecipitated with the corresponding specific antibody and protein A/G beads at 4 °C overnight with gentle agitation by vertical rotation. The immunoreactive products were washed three times and boiled in protein loading buffer for 10 min prior to SDS–PAGE. Bound proteins were detected using Western blotting as described above.

### GST Pulldown Assay

GST fusion proteins or His fusion proteins were expressed in E. coli BL21. To purify GST or GST‐tagged proteins, cells were lysed by sonication in lysis buffer (137 mm NaCl, 20 mm Tris‐HCl, 2 mm EDTA, 10% glycerol, and 1% Nonidet P‐40) supplemented with 1 mm PMSF and were then purified using Pierce Glutathione Agarose (Thermo, cat# 16101) at 4 °C for 1 h. The bead‐bound GST fusion proteins were washed with lysis buffer and were then incubated with His‐tagged protein extraction solution at 4 °C for 3 h with gentle agitation by vertical rotation. After unbound proteins were removed with washing buffer, bound proteins were eluted by boiling and separated via SDS–PAGE.

### Luciferase Reporter Assay

Cells were seeded in 24‐well plates and cotransfected with the STAT3 luciferase reporter hSIE/APRE (containing a STAT3‐responsive element), β‐galactosidase (β‐gal) expression vectors, and other relevant plasmids. After transfection, cells were treated with or without IL‐6 (25 ng mL, 6 h) for STAT3 activation. A luciferase assay was performed as described previously^[^ ^49^ ^]^, and luciferase activity was normalized to the corresponding β‐gal level.

### Chromatin Immunoprecipitation (ChIP) Assay

The ChIP assay was performed using a ChIP Assay Kit (Beyotime, cat# P2078) according to the manufacturer's instructions. In brief, cells were plated in 10‐cm dishes and fixed with 1% paraformaldehyde at 80–90% confluence. After the cells were lysed with SDS lysis buffer, the lysates were sonicated to generate 400–1000‐bp DNA fragments and immunoprecipitated using specific antibodies (4 µg of an anti‐STAT3 antibody and 8 µg of an anti‐OVOL2 antibody). Transcription factor binding‐ sites were predicted using the online *JASPAR* database (http://jaspardev.genereg.net). The STAT3 binding site sequences in CPT1A is: 5’‐cggtttttcctggaataaacta‐3’. The STAT3 binding site sequences in CPT1B is: 5’‐gctcacttccgggtaggagccc‐3’. The OVOL2 binding site sequence in JAK1 is 5’‐tattatagccgttacaattatt‐3’. The OVOL2 binding site sequence in JAK2 is 5’‐tccacaaaaactgctaggattt‐3’. The OVOL2 binding site sequence in TYK2 is 5’‐taacaattatgttattcttgtt‐3’. DNA products were analyzed using real‐time PCR and normalized to input DNA.

### DNA Affinity Precipitation Assay

Nuclear extracts (200 µg) were combined with 500 µL of DNA affinity precipitation assay buffer (25 mmol/l HEPES, pH 7.6, 60 mmol L^−1^ KCl, 5 mmol L^−1^ MgCl_2_, 7.5% glycerol, 0.1 mmol L^−1^ EDTA, 1 mmol L^−1^ dithiothreitol, and 0.25% Triton X‐100). This mixture included 0.2 nmol L^−1^ biotin‐labeled DNA probe and 10 µg poly (deoxyinosine‐deoxycytosine) [poly(Di‐Dc)] (Roche, cat# 10108812001) and was incubated overnight at 4 °C. Following this, 20 microliters of streptavidin magnetic beads (Beyotime, cat# P2151‐1 mL) were added, and the combination was further incubated for 2 h. After three washes with DNA affinity precipitation assay buffer, the resulting precipitates were subjected to Western blot analysis.

The sequence information for the probes used in this study is as follows:

CPT1A Probe:

ACATAAGGGATAGTTTATTCCAGGAAAAACCGAGCTTC

CPT1B Probe:

GGCTCCTACCCGGAAGTGAGCCAG

CON Probe:

TTCCCCGGGGAAAAATTTTCCCCGG

### ChIP Re‐IP Assay

Re‐Chromatin immunoprecipitation was conducted to confirm the coexistence of proteins and DNA within the same complex. Following an initial overnight immunoprecipitation using STAT3 antibody (1:100, CST), the protein‐DNA‐bead complexes underwent three washes with chromatin immunoprecipitation wash buffer, followed by a double wash with 1x TE buffer. Subsequently, the washed immunoprecipitated protein‐DNA complexes were eluted by incubating for 30 min at 37 °C in 75 µL of re‐chromatin immunoprecipitation elution buffer (2% sodium dodecyl sulfate, 0.1 mol L^−1^ NaHCO_3_), followed by centrifugation for 3 min at 600 g in a microcentrifuge to collect the beads at the bottom of the Eppendorf tube. After centrifugation, the supernatant was isolated and diluted 20 times (to a final volume of 1.5 mL) with chromatin immunoprecipitation dilution buffer supplemented with 50 µg of bovine serum albumin and protease inhibitors. A second immunoprecipitation reaction was then carried out using antibodies against HDAC1(1:200, Abcam) and OVOL2 (1:100, Novus).

### Statistical Analysis

GraphPad Prism 8.0 software (GraphPad Software Inc., CA, USA) was used to evaluate the data. The Kaplan–Meier method was used for tumor‐free survival and overall survival analysis. The two‐tailed Student's *t*‐test was performed to compare differences between groups, while analysis of variance (ANOVA) was used for comparisons among multiple groups. *p *< 0.05 was considered statistically significant.

### Ethics Approval and Consent to Participate

All animals were maintained in the Laboratory Animal Center at Xiamen University (China) in accordance with institutional guidelines. All mouse protocols and experiments were approved by the Animal Ethics Committee of Xiamen University (acceptance no. XMULAC20190043). All samples were collected between 15:00 and 17:00 to avoid circadian variability.

## Conflict of Interest

The authors declare no conflict of interest.

## Author Contributions

R.‐P.L. performed conceptualization, methodology, validation, formal analysis, data curation, visualization, and wrote—original draft. J.‐J.H. performed resources, investigation, data curation, and validation. T.F. performed resources, investigation, data curation, and validation. Y.Z. performed Investigation. R.‐Q.T. performed resources, investigation, and data curation. D.A. performed resources and investigation. S.W. performed an Investigation. Q.‐S.H. performed investigation. C.C. performed supervision. Z.‐M.Z. performed supervision, project administration, and funding acquisition. R.Z. performed supervision. H.G. performed conceptualization, wrote—reviewed, and edited, supervision, project administration, and funding acquisition. B.‐A.L. performed conceptualization, wrote—reviewed, and edited, supervision, project administration, and funding acquisition. All authors reviewed the manuscript. Further information and requests for resources and reagents should be directed to and will be fulfilled by the lead contact, Bo‐An Li (bali@xmu.edu.cn.)

## Supporting information

Supporting Information

## Data Availability

The data that support the findings of this study are available from the corresponding author upon reasonable request.
