# Supplementary material for: Loss of OVOL2 in Triple‐Negative Breast Cancer Promotes Fatty Acid Oxidation Fueling Stemness Characteristics
Source: Adv Sci (Weinh). 2024 Apr 16;11(24):2308945. doi: 10.1002/advs.202308945 (PMC11199980; doi:10.1002/advs.202308945)
Supplement: Supplementary file 1 — Supporting Information [file ADVS-11-2308945-s001.pdf]

ADVANCED SCIENCE
Open Access

Supporting Information

for *Adv. Sci.*, DOI 10.1002/advs.202308945

Loss of OVOL2 in Triple-Negative Breast Cancer Promotes Fatty Acid Oxidation Fueling Stemness Characteristics

Ruipeng Lu, Jingjing Hong, Tong Fu, Yu Zhu, Ruiqi Tong, Di Ai, Shuai Wang, Qingsong Huang, Ceshi Chen, Zhiming Zhang, Rui Zhang, Huiling Guo* and Boan Li**

Supplementary data

Loss of OVOL2 in triple-negative breast cancer promotes fatty acid oxidation fueling stemness characteristics

Ruipeng Lu[1#], Jingjing Hong[1#], Tong Fu[1#], Yu Zhu[1], Ruiqi Tong[1], Di Ai[1], Shuai Wang[1], Qingsong Huang[1], Ceshi Chen[4,5], Zhiming Zhang[3], Rui Zhang[2*], Huiling Guo [1*], Boan Li[1*]

This file includes:

- Supplementary Figures 1-7
- Supplementary Tables 1
- List of abbreviations

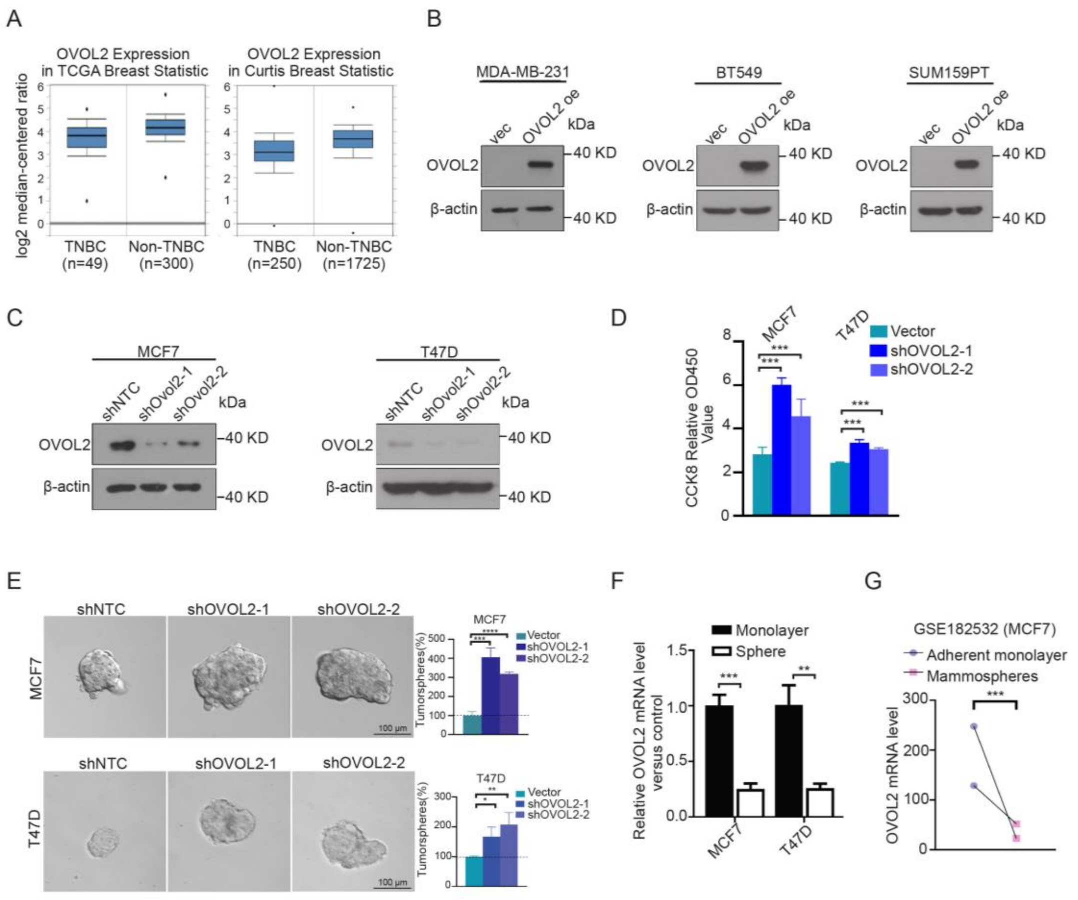

Figure S1. Enhancement of breast sphere formation by OVOL2 knockdown.

(A) Boxplots depicting the expression levels of OVOL2 in the TCGA breast cancer dataset and the Curtis breast cancer dataset from the Oncomine database. (B and C) Validation of OVOL2 protein overexpression or deficiency by Western blotting. (D) Cell growth was evaluated using a CCK8 assay. Data were analyzed 48 h after transfection. (E) The ability of OVOL2-knockdown cancer cells to induce mammosphere formation was determined by a mammosphere formation assay. The graphs show the sphere numbers compared to those in the control group. (F) Decreased OVOL2 mRNA expression in mammospheres formed from breast cancer cells. (G) The expression levels of OVOL2 in MCF7 adherent cells and breast sphere cells were analyzed based on the GSE databases (GSE182532). p-value determined by Student's t-test. The data

are presented as means ± SD. The symbols *, **, *** and represent P<0.05, P<0.01 and P<0.001, respectively.

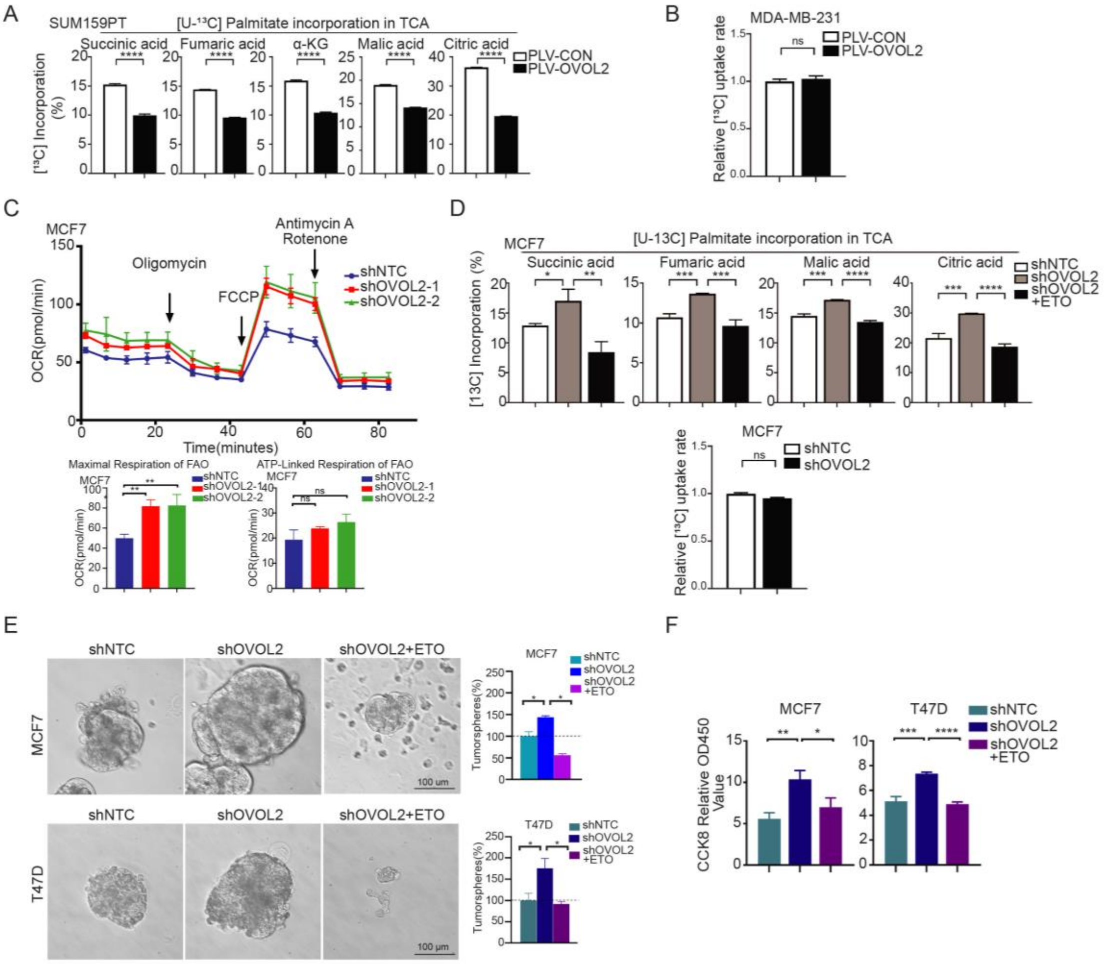

Figure S2. Knockdown of OVOL2 promotes sphere formation by enhancing FAO.

(A) Evaluation of $^{13}C_{16}$ palmitate incorporation using GCMS. (B) OVOL2 overexpression had no significant effect on fatty acid uptake. U-$^{13}C_{16}$ palmitate uptake, serving as an indicator of fatty acid uptake, was assessed. (C) FAO was evaluated by measuring oxygen consumption rate using palmitate-BSA as a substrate, following the methodology described in the Methods section. (D) Upper panel: Treatment with ETO compromised the OVOL2 knockdown-induced increase in U-$^{13}C_{16}$ palmitate incorporation into TCA intermediates. Down panel: OVOL2 knockdown had no

significant effect on fatty acid uptake. (E-F) Treatment with ETO compromised the OVOL2 knockdown-induced increases in mammosphere formation (E) and cell proliferation (F). p-value determined by Student's t-test. The data are presented as means ± SD. The symbols *, **, *** and represent P<0.05, P<0.01 and P<0.001, respectively.

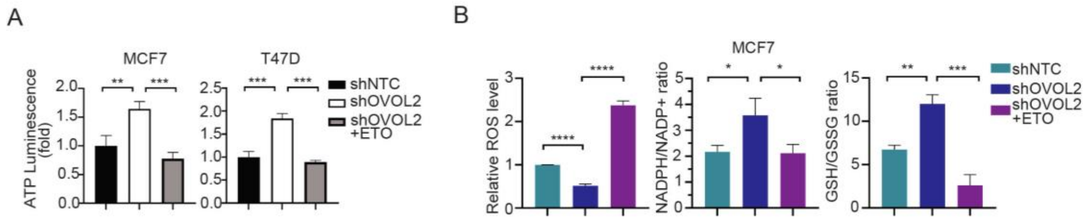

Figure S3. Effect of ETO treatment on the OVOL2 knockdown-induced alterations in ATP levels and ROS levels.

(A) Cellular ATP levels measured using an ATP assay kit. OVOL2 knockdown was performed in MCF7 cells, followed by treatment with ETO prior to the ATP assay. (B) Treatment with ETO compromised the OVOL2 knockdown-induced reduction in ROS levels and increases in the NADPH/NADP+ and GSH/GSSG ratios. p-value determined by two-way ANOVA test. The data are presented as means ± SD. The symbols *, **, *** and represent P<0.05, P<0.01 and P<0.001, respectively.

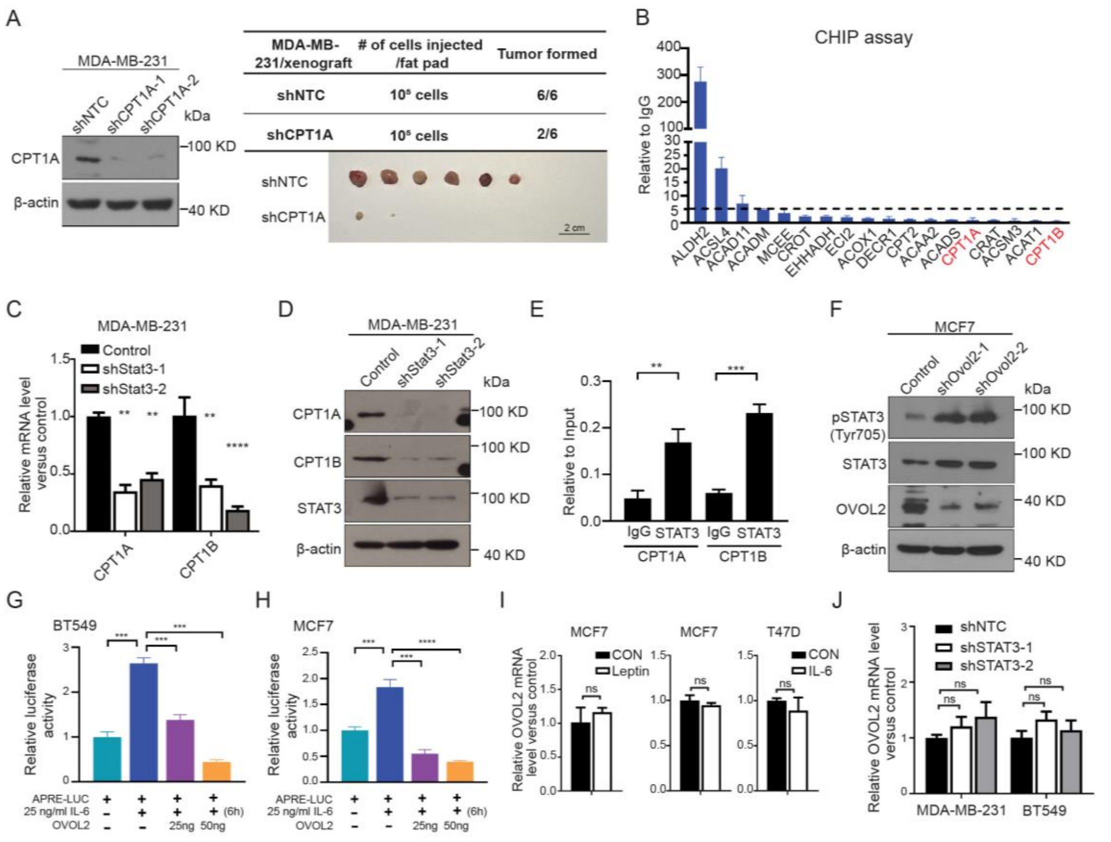

Figure S4. Regulation of STAT3 activity by OVOL2, with No reciprocal effect of STAT3 on OVOL2.

(A) Left panel: Validation of CPT1A knockdown. Right panel: Orthotopic injection of 1 × 10^5 MDA-MB-231 cells with normal CPT1A expression or CPT1A knockdown into 6-week-old female nude mice (n = 6). Representative images of engrafted tumors are displayed. (B) ChIP assay demonstrating the absence of OVOL2 binding to the promoters of CPT1A and CPT1B in MDA-MB-231 cells. The data were normalized to the IgG pulldown DNA. (C and D) qRT–PCR and Western blot analysis to measure CPT1A and CPT1B levels in MDA-MB-231 cells transduced with control or STAT3 shRNA. (E) ChIP assay showing STAT3 binding to the CPT1A and CPT1B promoter in MDA-MB-231 cells. The values were normalized to input DNA. (F) Western blot analysis of OVOL2 and phosphorylated STAT3 (pSTAT3 Tyr705) in MCF7 cells stably

transduced with control or OVOL2 shRNA. (G and H) Co-transfection of BT549 cells (G) and MCF7 cells (H) with the APRE-luciferase reporter and β-galactosidase plasmids in the presence of IL-6 (25 ng/ml) stimulation and transduction of the OVOL2-encoding vector. (I) qRT-PCR analysis demonstrated that Leptin (200 ng/ml) and IL-6 stimulation do not affect OVOL2 expression levels. (J) qRT–PCR was performed to measure OVOL2 mRNA levels in MDA-MB-231 and BT549 cells transduced with control or STAT3 shRNA. p-value determined by two-way ANOVA test. The data are presented as means ± SD. The symbols *, **, *** and represent P<0.05, P<0.01 and P<0.001, respectively.

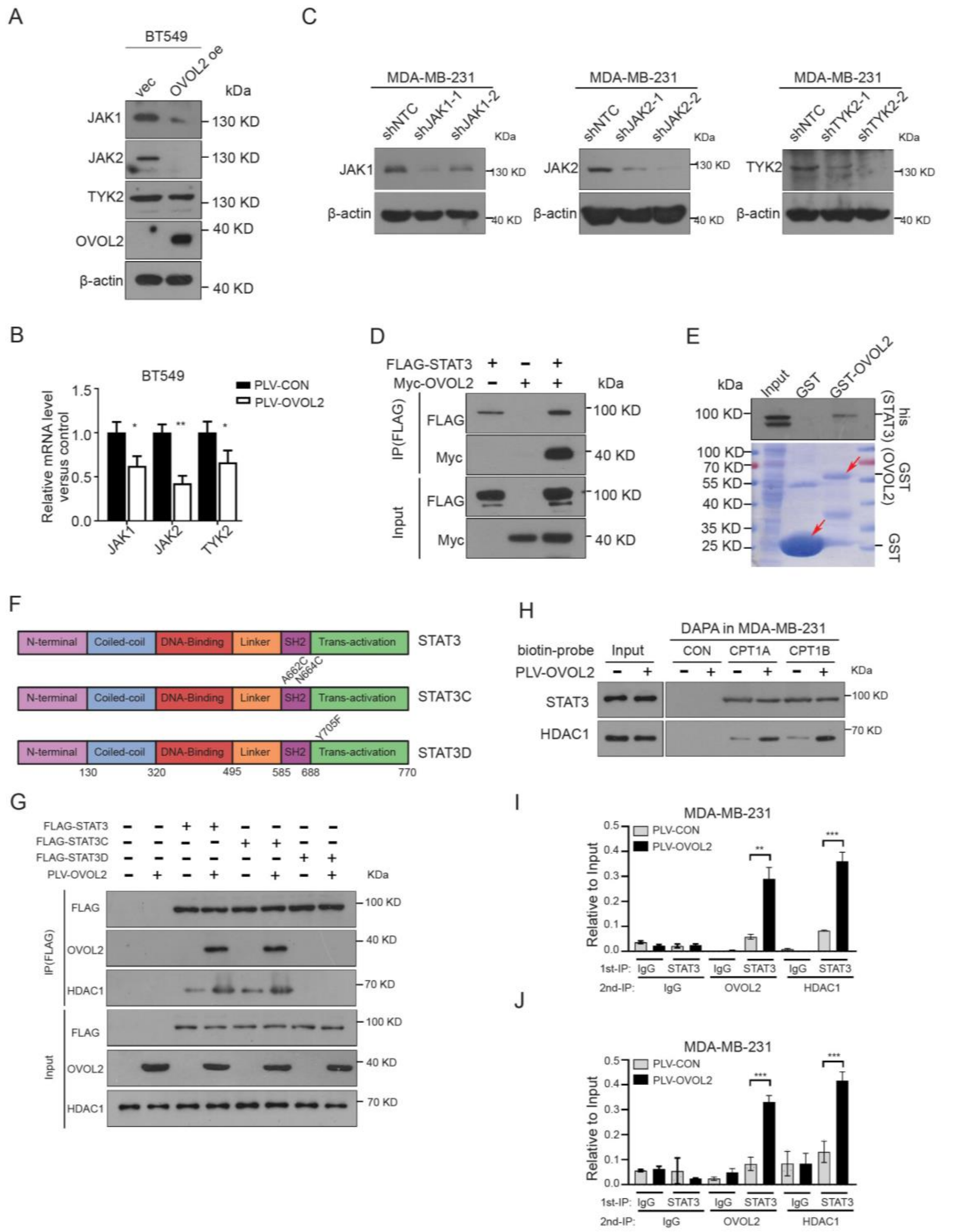

Figure S5. OVOL2 suppresses STAT3 phosphorylation and interacts with STAT3.

(A and B) Western blotting and qRT-PCR were used to measure the expression levels of JAK1, JAK2, and TYK2 upon OVOL2 overexpression in BT549 cells. (C) Western blot validation of JAK1, JAK2, and TYK2 silencing using two different shRNAs. (D) Co-IP of OVOL2 and STAT3, demonstrating their interaction. Whole-cell lysates were

subjected to IP using an anti-Flag antibody. (E) In vitro pulldown assay using GST-tagged OVOL2 and His-tagged STAT3, revealing direct binding between STAT3 and OVOL2. Target bands are indicated by red arrows. (F) Map of STAT3, STAT3C, STAT3D structure and mutation sites. (G) Co-IP experiments of STAT3-WT, STAT3C, and STAT3D with HDAC1 in OVOL2 overexpressed MDA-MB-231 cells. Whole-cell lysates were used for IP with an anti-Flag antibody. (H) OVOL2 enhances the association between HDAC1 and STAT3 binding site on the CPT1A and CPT1B promoter. MDA-MB-231 cells were infected with STAT3C and then processed for DNA affinity precipitation assay (DAPA). Probes containing biotin-labeled sequences corresponding to the STAT3 binding sites were used to enrich the DNA fragments along with the bound protein complexes. The captured DNA-protein complexes were then analyzed by Western blotting after being immobilized on streptavidin magnetic beads. (I and J) OVOL2 was overexpressed by lentivirus in MDA-MB-231 cells and subjected to sequential chromatin immunoprecipitation (re-ChIP) assay. The probes and primers were designed according to the CPT1A promoter sequence (I) and CPT1B promoter sequence (J). The first round of IP enriched the promoter sequences bound by STAT3, while the second round of IP investigated the binding of OVOL2 or HDAC1 to the promoter sequences bound by STAT3. p-value determined by Student's t-test. The data are presented as means ± SD. The symbols *, **, *** and represent $P<0.05$, $P<0.01$ and $P<0.001$, respectively.

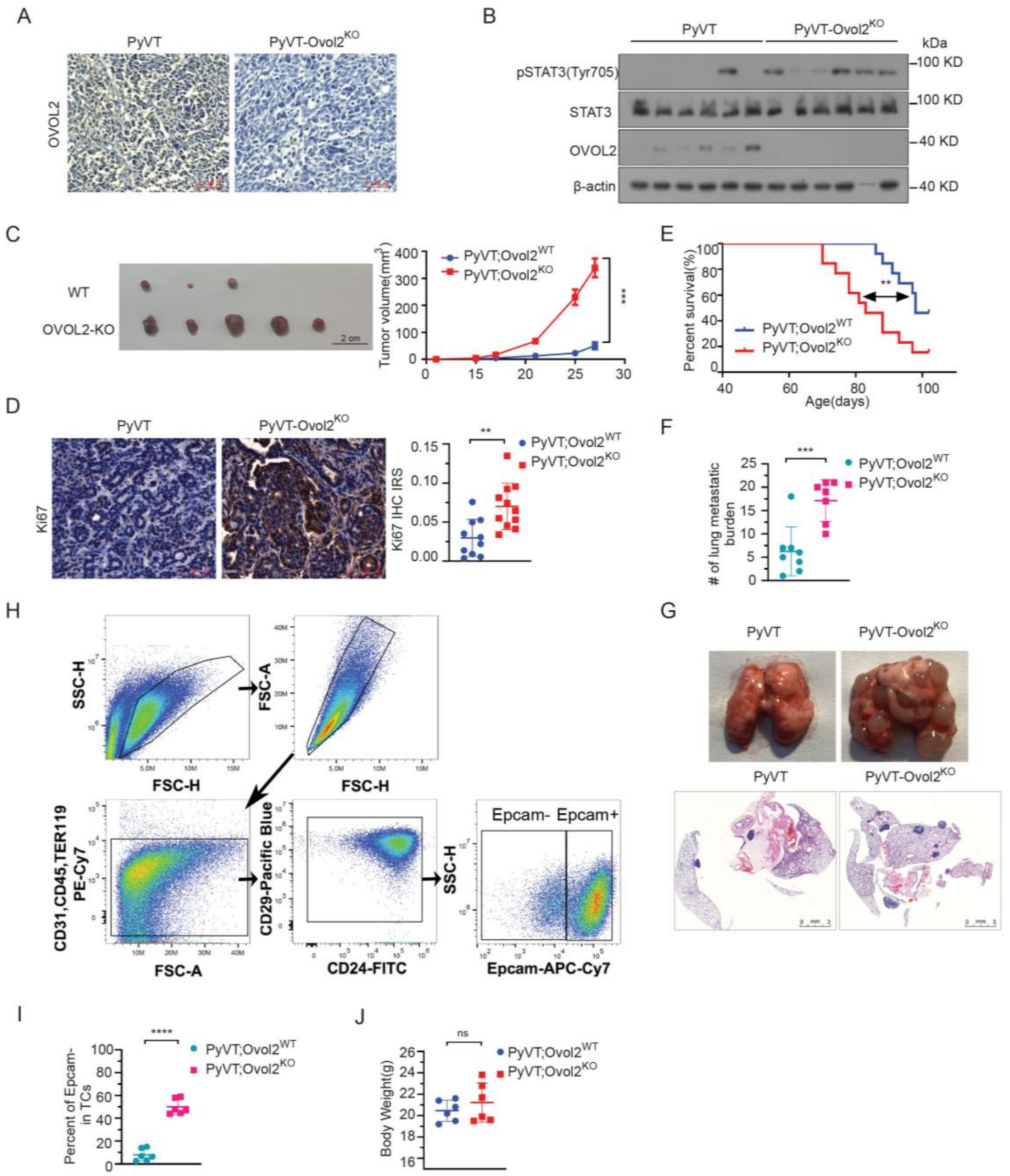

Figure S6. Loss of OVOL2 promotes tumor growth and metastasis.

(A) Immunostaining validation of OVOL2 expression in PyVT-OVOL2 knockout mice. Scale bars, 50 μm. (B). Western blot analysis of p-STAT3 and OVOL2 levels in tumor tissues. (C) Left: Representative images of tumors. Right: Tumor volume curve. (D) Evaluation of proliferation status by Ki67 IHC staining in mammary tumors. Tumors were harvested two weeks after tumorigenesis. (E) Kaplan-Meier plots showing overall survival in female PyVT (n = 13) and PyVT-OVOL2^{KO} (n = 13) mice. (F) Quantification

of lung metastatic foci (n = 7-8). Mice were sacrificed at 14 weeks. (G) Representative photographs of lung tissue sections stained with hematoxylin and eosin. (H) Flow cytometry plots illustrating the gating strategy used to isolate Epcam+ and Epcam- cells from MMTV-PyVT mammary tumors. (I) Determination of the percentage of Epcam- cells among isolated mammary tumor cells (n = 6). (J) Comparison of body weights between the two experimental groups of mice. The data are presented as means ± SEMs. Statistical significance is represented as ** ($P < 0.01$), *** ($P < 0.001$), **** ($P < 0.0001$), ns (not significant), determined by the Gehan-Breslow-Wilcoxon test.

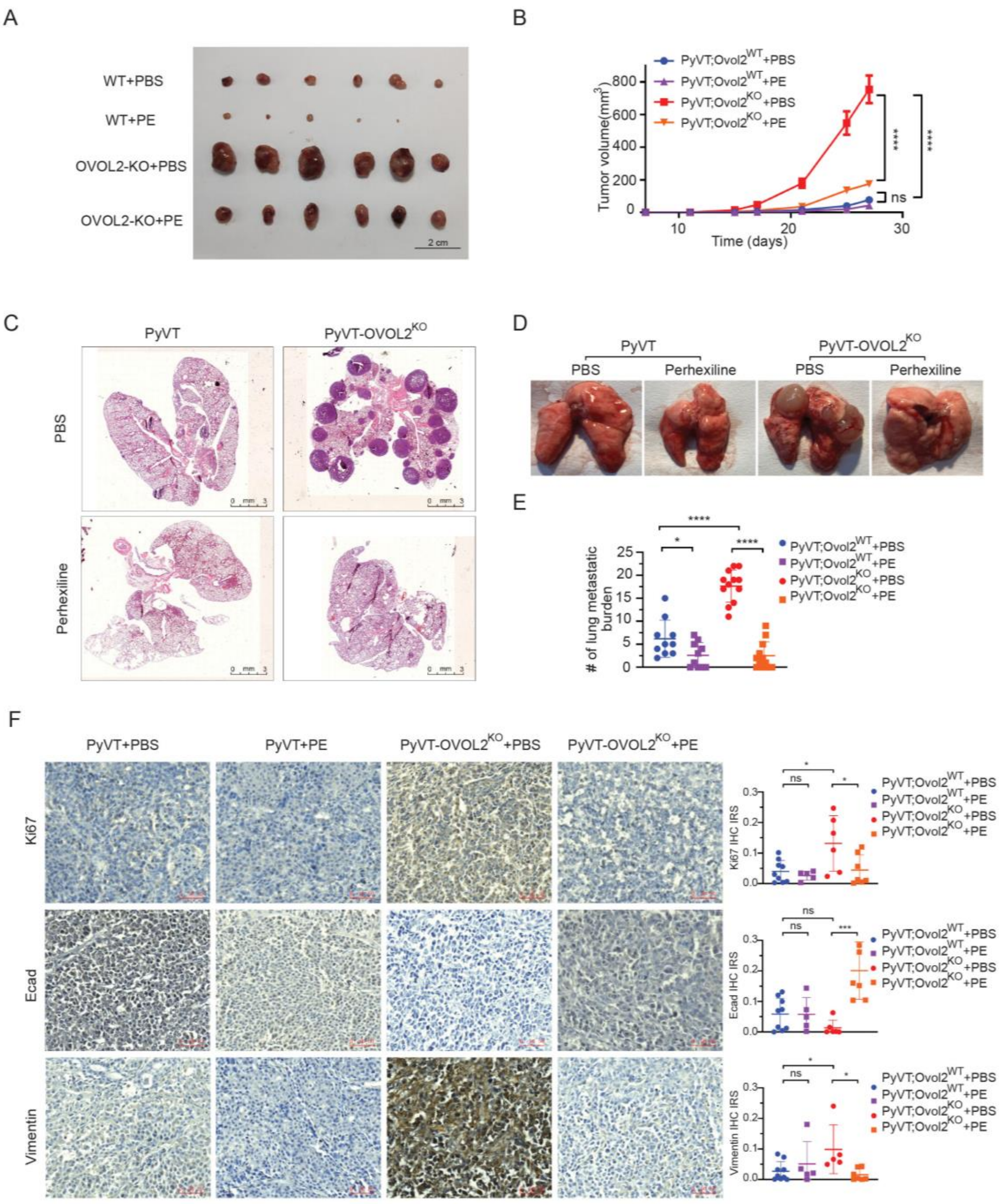

Figure S7. Regulation of tumor growth and metastasis by OVOL2 through FAO.

(A) Representative images of tumors. (B) Tumor growth curve. (C-E) Quantification of lung metastatic foci and representative pictures of metastases on the lung surface after different treatments. (F) IHC staining of Ki67, Ecad, and Vimentin in mammary tumors from MMTV-PyVT mice and PyVT-OVOL2^{KO} mice treated with vehicle control (PBS) or perhexiline. The data are presented as means ± SEMs. Statistical significance is

represented as ** (P < 0.01), *** (P < 0.001), **** (P < 0.0001), ns (not significant), determined by the Gehan-Breslow-Wilcoxon test.

Table S1. List of qPCR primer sequences.

* Primer	* Sequence（5'-3'）
18S qPCR F	GTCTGTGATGCCCTTAGATG
18S qPCR R	AGCTTATGACCCGCACTTAC
Ovol2 qPCR F	ACAGGCATTCGTCCCTACAAA
Ovol2 qPCR R	CGCTGCTTATAGGCATACTGC
STAT3 qPCR F	ACCAGCAGTATAGCCGCTTC
STAT3 qPCR R	GCCACAATCCGGGCAATCT
CPT1A qPCR F	ATCAATCGGACTCTGGAAACGG
CPT1A qPCR R	TCAGGGAGTAGCGCATGGT
CPT1B qPCR F	GCGCCCCTTGTTGGATGAT
CPT1B qPCR R	CCACCATGACTTGAGCACCAG
CPT2 qPCR F	CTGGAGCCAGAAGTGTTCCAC
CPT2 qPCR R	AGGCACAAAGCGTATGAGTCT
CRAT qPCR F	CAGGAAGACGGAGAACTGGC

CRAT qPCR R	CCTTGAAATCCAACACACCCTC
CROT qPCR F	GAAATTGGCTGGAAGAGTGGTG
CROT qPCR R	GAGTATTTCCAACTTTATGAACAGGC
ACAA2 qPCR F	CCAGCTCTCACGATTAATAGGCT
ACAA2 qPCR R	GGAGCTGGACATGCTGATCTGT
ACAT1 qPCR F	GCAGGCTTACCTATTTCTACTCCA
ACAT1 qPCR R	ACATGCTCTCCATCCCACCT
ACAD11 qPCR F	CAGGGTCGAATCTTCCGTGA
ACAD11 qPCR R	TTAGCCACTCCGATAGCTGTTG
ACADM qPCR F	ACAGGGGTTCAGACTGCTATT
ACADM qPCR R	TCCTCCGTTGGTTATCCACAT
ACADS qPCR F	CTCATCCACGGCCAACCTC
ACADS qPCR R	AGCGTAGTTCACAGCACAATCG
ACSL4 qPCR F	GGCTTCCTATCTGATTACCAGTGTT
ACSL4 qPCR R	GAATCTCAAATCCTTCAGGGTACTC
ACOX1 qPCR F	CATAATTTCATCAGGGTCAGCGA
ACOX1 qPCR R	ATTCCGCCAGCTTCAACCC
ACOX2 qPCR F	GAGTGTCATTGGGGGATACCTG
ACOX2 qPCR R	TCCGCATGGCAGCCTTATA
ACSM3 qPCR F	TCAAAACTCCATCGCATCTCTT
ACSM3 qPCR R	ACAGGACTTCAAACTGGGGATT

ACSM4 qPCR F	TGGCAGCTTTTCGGGACA
ACSM4 qPCR R	CTGACTTTGAAGCCATAAATCGC
EHHADH qPCR F	CCCTGCAATATGCTTTCTTCG
EHHADH qPCR R	GCCGCTCTGTTGCATTTTG
ALDH2 qPCR F	ATGGCAAGCCCTATGTCATCT
ALDH2 qPCR R	CCGTGGTACTTATCAGCCCA
DECR1 qPCR F	CTATGCTGAGACTGGTTCAGGT
DECR1 qPCR R	CCAGACGGCTAAAGGCACC
ECI2(PECI) qPCR F	CATTCCATGCGTCCCATTTG
ECI2(PECI) qPCR R	GAATAGAACAGCAATGAGAGCCAG
MCEE qPCR F	AAACAACAGATACTCCATGTTCAGG
MCEE qPCR R	TGCCGTAGGGCTTTTTTCC
JAK1 qPCR F	CTTTGCCCTGTATGACGAGAAC
JAK1 qPCR R	ACCTCATCCGGTAGTGGAGC
JAK2 qPCR F	AAACGGTGGAATTCAGTGGTCA
JAK2 qPCR R	TGCTTATGGATAGTTACAACTCGGC
JAK3 qPCR F	TTCGGGCTACGCAAGGATTTG
JAK3 qPCR R	AGGCTGAGACACTCACCCT
TYK2 qPCR F	GAGATGCAAGCCTGATGCTAT
TYK2 qPCR R	GGTTCCCGAGGATTCATGCC

List of abbreviations

ATCC	American Type Culture Collection
BSA	Bovine serum albumin
CCK8	Cell Counting Kit-8
ChIP	Chromatin immunoprecipitation
Co-IP	Co-immunoprecipitation
CPT1A	Carnitine palmitoyl transferase 1A
CPT1B	Carnitine palmitoyl transferase 1B
CSCs	Cancer stem cells
DAPA	DNA affinity precipitation assay
DMEM	Dulbecco's modified Eagle's medium
EMT	Epithelial-mesenchymal transtition
ER	Estrogen receptor
ETO	Etomoxir
FABP4	Fatty acid-binding protein 4
FACS	fluorescence-activated cell sorting
FAO	Fatty acid oxidation
FAO-OCR	FAO-specific oxygen consumption rate
GEO	Gene Expression Omnibus
GSH/GSSG	Glutathione/oxidized glutathione
HER2	Human epidermal growth factor receptor 2

H&E	Hematoxylin and eosin
IHC	Immunohistochemical
IL-6	Interleukin-6
IL-8	Interleukin-8
IP	Immunoprecipitation
IRSs	Immunoreactive scores
KO	Knockout
NAC	N-acetylcysteine
OCR	oxygen consumption rates
OVOL2	Ovo-like 2
PE	perhexiline
PEI	Polyethylenimine
pLV	pBOBi lentiviral
PR	Progesterone receptor
RNA-Seq	RNA sequencing
ROS	Reactive oxygen species
RT-qPCR	Quantitative reverse transcription-PCR
SNAG domain	Snail/Gfi-1 domain
TCA cycle	Tricarboxylic acid cycle
TIC	Tumour-initiating capacity
TNBC	Triple-negative breast cancer
Tyr	Tyrosine

Upa	Upadacitinib
WT	Wild type
β-gal	β-galactosidase
